# Core–Shell Crystals of Porous Organic Cages

**DOI:** 10.1002/anie.201803244

**Published:** 2018-06-10

**Authors:** Shan Jiang, Yi Du, Marco Marcello, Edward W. Corcoran, David C. Calabro, Samantha Y. Chong, Linjiang Chen, Rob Clowes, Tom Hasell, Andrew I. Cooper

**Affiliations:** ^1^ Department of Chemistry, Materials Innovation Factory University of Liverpool Liverpool L69 7ZD UK; ^2^ Corporate Strategic Research ExxonMobil Research and Engineering Company 1545 U.S. Highway 22 Annandale NJ 08801 USA; ^3^ Institute of Integrative Biology University of Liverpool Crown Street Liverpool L69 7ZD UK

**Keywords:** adsorption selectivity, core–shell crystals, porous cage crystals, surface hydrophobicity

## Abstract

The first examples of core–shell porous molecular crystals are described. The physical properties of the core–shell crystals, such as surface hydrophobicity, CO_2_ /CH_4_ selectivity, are controlled by the chemical composition of the shell. This shows that porous core–shell molecular crystals can exhibit synergistic properties that out‐perform materials built from the individual, constituent molecules.

The preparation of new functional porous materials is an important goal in materials chemistry, with potential applications in gas storage, molecular separations, catalysis, and sensing.^1^ Established classes of porous materials include extended networks and frameworks such as zeolites,^2^ metal‐organic frameworks (MOFs),^3^ covalent organic frameworks (COFs),^4^ and crosslinked polymers.^5^ More recently, porous molecular solids have emerged as a new materials platform.^6^ There has been much effort to increase the structural complexity of porous solids to create materials with differentiated or multiple functions, such as mixed‐component MOFs^7^ and epitaxial MOF thin films.^8^ Another approach is to create core–shell porous materials that can integrate multiple functionalities into the core and shell layers.^9^

Core–shell MOF structures can be formed via strong coordination bonds where the outer shell layer is grown epitaxially on the surface of an inner MOF core.^10^ This way, the overall material properties can be enhanced by combining different functionalities in the core and shell layers.^11^ For example, the integration of a shell crystal with selective gas sorption with a core crystal with high pore volume makes it possible to combine gas selectivity with high gas storage capacity.^12^ Also, core–shell nanostructures with an inner core nanoparticle encapsulated by a porous shell have been widely used for heterogeneous catalysis, where the shell material can ensure the accessibility of reactant molecules to the active metal and also improve the selectivity and stability of the catalyst.^13^ However, it remains challenging to incorporate functionality in three‐dimensional (3D) core–shell porous structures in a modular way, ideally via a simple solution process. The fabrication of core–shell porous solids with a defect‐free, crack‐free shell layer is also still a challenge.

We have developed a series of porous organic cages (POCs) with properties such as shape‐specific molecular sieving,^14^ underpinned by computational design methods such crystal structure prediction.^15^ A distinguishing feature of POCs is that they can be dissolved in common solvents. This enables a range of processing options that are not available to porous extended networks. For example, cage nanoparticles can be prepared by mixing cage molecules of opposite chirality in solution.^16^ Mix‐and‐match assembly strategies can also be used to make binary and ternary cocrystals.^17^

Here, we develop a simple and efficient method to assemble core–shell POC nanostructures in a modular manner. The synthesis involves the sequential addition of solutions of the *R* and *S* cage enantiomers that exploits chiral recognition. This solution‐based mixing process yields core–shell cocrystals with exceptional control over particle size and morphology, also allowing control over surface hydrophobicity. Moreover, CO_2_/CH_4_ selectivity can be tuned by varying the gas selectivity of the defect‐free particle shell. To our knowledge, this is the first example of porous molecular core–shell materials.

The POC molecules were synthesized via [4+6] cycloimination reactions.^18^ **CC3**‐*R* (Figure 1 a, left) was synthesized from 1, 3, 5‐triformylbenzene (TFB) and (1*R*, 2*R*)‐(−)‐1,2‐diaminocyclohexane (*R*, *R*‐CHDA).15a In the CC3a crystal form **CC3**‐*R* packs in a window‐to‐window fashion to create 3D diamondoid pores connected through the internal cage voids (Brunauer–Emmett–Teller surface area, SA_BET_, 409 m^2^ g^−1^; Figure 1 c, left).^18^ The opposite **CC3** cage enantiomer can be formed using (1*S*, 2*S*)‐(−)‐1,2‐diaminocyclohexane (*S*, *S*‐CHDA).

**Figure 1:**
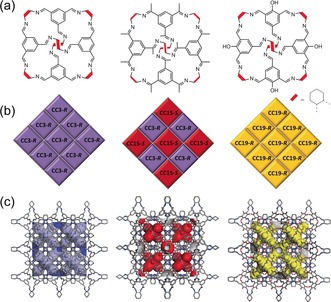
a) Organic cage molecules; **CC3** (left), **CC15** (middle) and **CC19** (right). b) Scheme showing the window‐to‐window packing of homochiral cages (**CC3**‐*R* and **CC19**‐*R*) and a quasi‐racemic cocrystal of **CC3**‐*R* and **CC15**‐*S*. c) Connolly surface area generated using a N_2_ probe radius of 1.82 Å to show 3D diamondoid interconnected pore structures for **CC3**‐*R* (left), a cocrystal of **CC3**‐*R* and **CC15**‐*S* (middle), and **CC19**‐*R* (right).

An analogous [4+6] cage molecule, **CC15**‐*R*, can be synthesized from 1,3,5‐triacetylbenzene (TAB) and *R*, *R*‐CHDA. **CC15**‐*R* has 12 methyl groups positioned in the windows of the cage (Figure 1 a, middle).15b By itself, **CC15**‐*R* does not show the preferred window‐to‐window packing that is observed for **CC3**‐*R* because of steric interactions between these methyl groups. However, a quasiracemic cocrystal of **CC3**‐*S* and **CC15**‐*R* does pack in a window‐to‐window fashion (Figure 1 c, middle), as rationalized previously by crystal structure prediction.15b Because the methyl groups in **CC15** partially block the cage windows, the (**CC3**‐*S*, **CC15**‐*R*) cocrystal becomes selectively porous to H_2_ but not N_2_ at 77 K, 1 bar.15b Another cage molecule with an analogous tetrahedral architecture, first reported by Petryk et al.,^19^ can be prepared by 2‐hydroxy‐1, 3, 5‐benzenetricarbaldehyde with *R*, *R*‐CHDA. We will refer to this covalent cage here as **CC19** (Figure 1 a, right). The disordered hydroxyl groups occupy the four cage windows. **CC19**‐*R* crystallizes to form a window‐to‐window packing with 3D diamondoid pores, isostructural with **CC3**α (Figure 1 c right). **CC19**‐*R* shows permanent porosity to a range of gases and exhibits a type I N_2_ sorption isotherm with a SA_BET_ of 514 m^2^ g^−1^ (Figure S2 in the Supporting Information).

Three different heterochiral cage compositions were used in this study: racemic **CC3**‐*RS*, racemic **CC19**‐*RS*, and quasiracemic **CC3**‐*R*, **CC15**‐*S*. In each case, cage particles were fabricated by simple mixing of the corresponding *R* and *S* solutions, taking advantage of the lower solubility product of the racemic or quasiracemic materials.^16^ All heterochiral cage particles were crystalline and each had the same basic packing mode, as demonstrated by powder X‐ray diffraction (PXRD) (Figure S3,4). The similar lattice parameters for the three different compositions suggested the potential for epitaxial growth to create core–shell structures. All cage particles showed uniform, octahedral crystal morphologies (e.g., Figure 2 b). The particle size could be controlled systematically in the range 250 nm to 2 μm by varying the mixing temperature (Figure S5). To probe the potential for core–shell structure generation, we first investigated the sequential addition of **CC3**‐*R* and **CC3**‐*S* solutions to see whether this would make larger particles by seeded, epitaxial growth, or whether new particles would be nucleated. The particle sizes measured by dynamic light scattering (DLS) and by scanning electron microscopy (SEM) for each addition confirmed that progressively larger particles were formed (Figure S6, Table S1), suggesting epitaxial growth and the possibility of core–shell structure generation by sequential addition of solutions of distinct cages.

**Figure 2:**
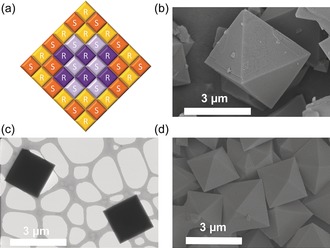
a) General scheme showing the structure of a core–shell multicomponent heterochiral cage cocrystals (core=purple/mauve; shell=yellow/orange). b) SEM image of a large **CC3**‐*RS*_core_/**CC19**‐*RS*_shell_ crystal. c,d) TEM and SEM images of large **CC19**‐*RS*_core_/**CC3**‐*RS*_shell_ crystals.

Next, we prepared core–shell structures using **CC3** and **CC19** cage molecules. The schematic structure is shown in Figure 2 a; the core molecules are colored purple. Two core–shell crystal systems were prepared: **CC3**‐*RS*_core_/**CC19**‐*RS*_shell_ and its inverse structure, **CC19**‐*RS*_core_/**CC3**‐*RS*_shell_, both using the sequential addition method described above using DCM solutions at 30 °C. The average DLS particle diameters for the core–shell cocrystals, **CC3**‐*RS*_core_/**CC19**‐*RS*_shell_ and **CC19**‐*RS*_core_/**CC3**‐*RS*_shell_, were 744 nm and 721 nm, respectively, as compared to 212 nm and 474 nm for the **CC3**‐*RS* and **CC19**‐*RS* core seed particles (Figure S7, Table S2). This would suggest a **CC19**‐*RS* shell thickness of 266 nm in **CC3**‐*RS*_core_/**CC19**‐*RS*_shell_ and a **CC3**‐*RS* shell thickness of 124 nm in **CC19**‐*RS*_core_/**CC3**‐*RS*_shell_. The particle size was further verified by SEM, as shown in Figure S8. There was a good agreement between the DLS and SEM measurements. Larger crystals were required to confirm the core–shell morphology by microscopy. We therefore mixed the solutions in CHCl_3_ at a higher temperature (50 °C), whereupon the average particle size of the core–shell crystals was increased to 3–4 μm, as shown in Figure 2 b–d: **CC3**‐*RS* (≈2 μm) and **CC19**‐*RS* (1–2 μm) prepared under the same conditions (Figure S9,10). A terraced surface structure was observed by SEM (Figure 2 b, Figure S11) indicating the epitaxial growth of the shell. The core–shell samples showed uniform octahedral shape morphologies without any apparent particle aggregation during the formation of the shell.

Since no contrast could be seen between the chemically‐similar core and shell by TEM (Figure 2 c), the morphologies of the **CC3**‐*RS*_core_/**CC19**‐*RS*_shell_ and **CC19**‐*RS*_core_/**CC3**‐*RS*_shell_ cocrystals were explored by confocal laser scanning microscopy (CLSM). This was possible because **CC19**‐*RS*, unlike **CC3**‐*RS*, is strongly fluorescent. To visualize the layered core–shell structure, we used ≈5 micrometer‐sized core–shell cocrystals prepared in CHCl_3_ at 60 °C. The horizontally sliced confocal image of **CC3**‐*RS*_core_/**CC19**‐*RS*_shell_ revealed a non‐fluorescent inner core (**CC3**‐*RS*) encapsulated by a fluorescent outer shell layer (**CC19**‐*RS*), as shown in Figure 3 c and the corresponding 3D structural model (Movie S1). By contrast, the **CC19**‐*RS*_core_/**CC3**‐*RS*_shell_ crystals comprise a non‐fluorescent **CC3**‐*RS* shell encapsulating a fluorescent core (**CC19**‐*RS*) (Figure 3 d). The intensity profiles are presented in Figure 3 e,f, which correspond to the core–shell crystals shown in the horizontally sliced images (Figure 3 c,d). The distance across the crystal is approximately 6 μm for **CC3**‐*RS*_core_/**CC19**‐*RS*_shell_, and this representative crystal has a non‐fluorescent core of approximately 3 μm in diameter and a 1.5 μm‐thick shell, as estimated from the fluorescence intensity profiles. The diameter of the **CC19**‐*RS*_core_/**CC3**‐*RS*_shell_ crystal was 4 μm with a 3 μm fluorescent core and a 500 nm non‐fluorescent shell. Z‐stack of CLSM images of **CC3**‐*RS*_core_/**CC19**‐*RS*_shell_ and **CC19**‐*RS*_core_/**CC3**‐*RS*_shell_ are shown in Figure S12,13. A 3D structural model for **CC3**‐*RS*_core_/**CC19**‐*RS*_shell_ was constructed based on the *z*‐stack of CLSM analysis (SI, Movie S2).

**Figure 3:**
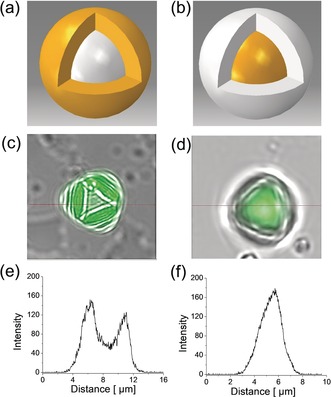
Schemes illustrating a) a **CC3**‐*RS*_core_/**CC19**‐*RS*_shell_ structure with a non‐fluorescent core (white) and the fluorescent shell (yellow) and b) a **CC19**‐*RS*_core_/**CC3**‐*RS*_shell_ structure with a fluorescent core (yellow) and a non‐fluorescent shell (white); c) Confocal laser scanning microscope (CLSM) image for **CC3**‐*RS*_core_/**CC19**‐*RS*_shell_; d) CLSM image for **CC19**‐*RS*_core_/**CC3**‐*RS*_shell_; Fluorescence intensity profiles for e) **CC3**‐*RS*_core_/**CC19**‐*RS*_shell_ and f) for **CC19**‐*RS*_core_/**CC3**‐*RS*_shell_.

The structural relationship between the core seed crystals, separate crystals of the shell components, and the core–shell cocrystals was further explored by synchrotron X‐ray diffraction. Both **CC3**‐*RS* and **CC19**‐*RS* crystallized in the cubic space group *F*4_1_32 with unit cell parameters of *a*=24.7069(1) Å for **CC3**‐*RS* and *a*=24.6914(3) Å for **CC19**‐*RS*. Lattice parameter matching is important in allowing the growth of the core–shell morphology. The PXRD patterns for **CC3**‐*RS*, **CC19**‐*RS*, and **CC3**‐*RS*_core_/**CC19**‐*RS*_shell_ (Figure S14) indicate that the core–shell particles retain a similar crystal packing: the core–shell cage crystals also crystallize with cubic symmetry and window‐to‐window packing motifs, analogous to **CC3**‐*RS* and **CC19**‐*RS*, with a small expansion in the unit cell parameters compared to the individual racemic crystals (Table S3).

**CC3**‐*RS*_core_/**CC19**‐*RS*_shell_ demonstrates a significantly higher oxygen content as measured by X‐ray photoelectron spectroscopy (XPS) due to an outer layer containing hydroxyl groups (oxygen elements), while **CC19**‐*RS*_core_/**CC3**‐*RS*_shell_ does not (Table S4). Also, the solution UV absorption spectrum for **CC19**‐*RS* shows absorption peaks at 300 and 375 nm. By contrast, a **CC3**‐*RS* solution exhibits no UV adsorption in this region. The absorption peaks for the core–shell, **CC3**‐*RS*_core_/**CC19**‐*RS*_shell_, as measured by dispersing the cage particles in the hexane suspension, showed a slight blue shift relative to the **CC19**‐*RS* solution spectrum, while a red shift was observed for the **CC19**‐*RS*_core_/**CC3**‐*RS*_shell_ material (Figure S16). The intensity of the fluorescence excitation/emission spectra for **CC19**‐*RS*_core_/**CC3**‐*RS*_shell_ was significantly decreased as compared to **CC19**‐*RS*, in keeping with a fluorescent core of **CC19**‐*RS* that is encapsulated by a non‐fluorescent **CC3**‐*RS* layer (Figure S17).

This synthetic method can also be applied to other cage molecules: for example, a core–shell crystal with racemic **CC3** as the core and quasi‐racemic **CC3**‐*R*/**CC15**‐*S* as the shell was also prepared. The **CC3**‐*RS* core crystals had an average particle size of 1–2 μm, as measured by SEM. Subsequent addition of solutions of **CC3**‐*R* and **CC15**‐*S* formed a shell, creating a **CC3**‐*RS*_core_/**CC15** *S*‐**CC3** *R*_shell_ cocrystals with an average diameter of 3 μm (Figure S18, S19).

Core–shell structures can be exploited to control particle surface properties, which are important in applications such as gas storage and separation.^20^ Contact angles with water for cage crystals (1–3 μm diameter) gradually increased from 55.68±2.5° (**CC19**‐*RS*) to 78.71±0.80° (**CC3**‐*RS*) to 83.06±3.04° (**CC3**‐*R*/**CC15**‐*S*) as the constituent cage materials become more hydrophobic (Figure S20). **CC3**‐*RS*_core_/**CC19**‐*RS*_shell_ shows a contact angle of 59.71±6.5°: that is, very close to the pure, relatively hydrophilic **CC19** material (Figure 4 a), showing that the shell dominates the surface properties. Likewise, the inverse **CC19**‐*RS*_core_/**CC3**‐*RS*_shell_ cocrystal showed a contact angle of 79.01±3.1°, close to pure **CC3**‐*RS*. The contact angle of **CC3**‐*RS*_core_/**CC15** *S*‐**CC3** *R*_shell_ is 83.40±0.87°; this material is slightly more hydrophobic due to the methyl groups in **CC15**.

**Figure 4:**
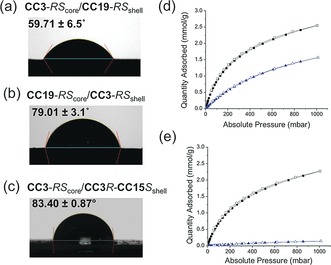
Contact angle measurement for a) **CC3**‐*RS*_core_/**CC19**‐*RS*_shell_, b) **CC19**‐*RS*_core_/**CC3**‐*RS*_shell_, and c) **CC3**‐*RS*_core_/ **CC15** *S*‐**CC3** *R*_shell_; CO_2_ and CH_4_ adsorption and desorption isotherms at 273 K for d) **CC19**‐*RS*_core_/**CC3**‐*RS*_shell_ and e) the inverse morphology, **CC3**‐*RS*_core_/**CC19**‐*RS*_shell_. CO_2_ isotherms shown as black squares, methane as blue triangles (closed symbols for adsorption, open for desorption).

Gas sorption analysis was carried out for both **CC19**‐*RS*_core_/**CC3**‐*RS*_shell_ and **CC3**‐*RS*_core_/**CC19**‐*RS*_shell_ core–shell materials. N_2_ sorption measurements at 77 K showed very similar Type I isotherms for both core–shell cage cocrystals (Figure S21). We found that CO_2_/CH_4_ selectivity was defined by the crystal shell. **CC19**‐*RS*_core_/**CC3**‐*RS*_shell_ was porous to both CO_2_ and CH_4_ at 273 K, 1 bar and had rather poor selectivity for these two gases (Figure 4 d). By contrast, **CC3**‐*RS*_core_/**CC19**‐*RS*_shell_ was selectively porous to CO_2_ under the same conditions (Figure 4 e). The ideal adsorbed solution theory (IAST) selectivity of **CC3**‐*RS*_core_/**CC19**‐*RS*_shell_ was 33, as calculated using experimental single‐component isotherms at 273 K with CO_2_/CH_4_ mixtures (50/50 molar ratio; see Figure S22b). This core–shell material combines a high capacity for CO_2_ (2.5 mmol g^−1^) with good CO_2_/CH_4_ selectivity. The high CO_2_ sorption capacity is attributed to the **CC3**‐*RS* core while the selectivity results from the **CC19**‐*RS* shell, which inhibits CH_4_ diffusion into the core. The **CC3**‐*RS*_core_/**CC19**‐*RS*_shell_ material therefore has synergistic properties that are not exhibited by the individual cage components, nor by the inverse **CC19**‐*RS*_core_/**CC3**‐*RS*_shell_ morphology, illustrating the power of this approach. A summary of gas sorption data is given in Table S4.

For practical application, it is preferable for core–shell crystals to be defect and crack free, since cracks in the shell layer could allow direct access to the core, reducing selectivity. Neither SEM nor TEM images revealed any obvious cracks on the cage particle surfaces (Figure S23). Moreover, core–shell crystals were immersed into a solution of a fluorescent organic dye (Rose Bengal) that would be size excluded from the cage pores but not from larger cracks or defects. For most crystals (approx. 90 %), horizontally sliced confocal images showed that most of the dye was coated onto the surface of the core–shell cage crystal (Figure S24), indicating that there were no significant cracks or defects in the shell layer. However, around 10 % of the crystals that we measured appeared to show some sort of mechanical damage, which might affect the adsorption properties (Figure S25).

In conclusion, we have successfully prepared core–shell cage crystals. The surface chemistry is controlled by the functionality in the shell layer, thus allowing control over surface hydrophobicity. Hence, **CC3**, which was shown previously to have multiple practical applications,^21^ can be rendered either more hydrophobic or more hydrophilic, depending on the choice of shell. A **CC3**‐*RS*_core_/**CC19**‐*RS*_shell_ material was shown to have a synergistic combination of CO_2_ sorption capacity and CO_2_/CH_4_ selectivity that surpassed either of individual constituent cages. This approach has the potential to open up new applications for porous organic cages. To give one example, **CC3** crystals have been incorporated into polymers of intrinsic microporosity to form organic mixed matrix membranes (MMMs) for molecular sieving.^22^ In MMMs, a good interaction between the polymer and filler components is essential, and this core–shell approach offers a new strategy for optimizing the polymer‐cage particle interface. It is also possible that cage shells could be deposited from solution onto porous crystals of other materials such as MOFs, COFs and zeolites, providing that conditions can be identified to promote epitaxial growth.

## Conflict of interest

The authors declare no conflict of interest.

## Supporting information

As a service to our authors and readers, this journal provides supporting information supplied by the authors. Such materials are peer reviewed and may be re‐organized for online delivery, but are not copy‐edited or typeset. Technical support issues arising from supporting information (other than missing files) should be addressed to the authors.

Supplementary

Supplementary

Supplementary
