# Supplementary material for: Core–Shell Crystals of Porous Organic Cages
Source: Angew Chem Int Ed Engl. 2018 Jun 10;57(35):11228–32. doi: 10.1002/anie.201803244 (PMC6120484; doi:10.1002/anie.201803244)
Supplement: Supplementary file 1 — Supplementary [file ANIE-57-11228-s001.pdf]

Angewandte Chemie
Eine Zeitschrift der Gesellschaft Deutscher Chemiker

Supporting Information

Core–Shell Crystals of Porous Organic Cages

Shan Jiang, Yi Du, Marco Marcello, Edward W. Corcoran, Jr., David C. Calabro, Samantha Y. Chong, Linjiang Chen, Rob Clowes, Tom Hasell, and Andrew I. Cooper**

anie_201803244_sm_miscellaneous_information.pdf
anie_201803244_sm_Movie_S1.mpg
anie_201803244_sm_Movie_S2.wmv

Supplementary Information

This file contains:

Methods

Characterizations
References

Supplementary text

Figures S1 to S25

Tables S1 to S5

Captions for Movies S1 to S2.

Other supplementary materials for this manuscript include:

Movies S1 to S2.

Table of contents

Figure S1. ^{1}H NMR spectrum ($CDCl_3$) of (a) **CC3**-*R*, (b) **CC15**-*R* and (c) **CC19**-*R*.

Figure S2. Gas sorption isotherm for N_2 at 77 K for **CC19**-*R*.

Figure S3. PXRD patterns for racemic **CC3**-*RS*, **CC19**-*RS*, and quasi-racemic **CC3**-*R*/**CC15**-*S*.

Figure S4. Scheme showing the cage packing of (a) racemic **CC3**-*RS*, (b) quasi-racemic **CC3**-*R*/**CC15**-*S* and (c) racemic **CC19**-*RS*.

Figure S5. (a) Particle size distributions of **CC3**-*RS* prepared at various mixing solution concentrations and in different solvent systems. (b) Particle size distributions of **CC19**-*RS* prepared at various mixing solution concentrations and in different solvent systems.

Figure S6. SEM images for racemic **CC3** particles prepared by the sequential addition of **CC3**-*R* and **CC3**-*S* solutions**.**

Table S1. Particle sizes for the racemic **CC3**-*RS* as prepared by the sequential addition method.

Figure S7. Particle size distributions from dynamic light scattering (DLS) for **CC3**-*RS*, **CC19**-*RS*, **CC3**-RS_{core}/**CC19**-RS_{shell} and **CC19**-RS_{core}/**CC3**-RS_{shell}. The samples were prepared in DCM solutions at 30 ˚C.

Table S2. Particle sizes calculated from DLS.

Figure S8. SEM images for **CC3**-RS_{core}/**CC19**-RS_{shell} and **CC19**-RS_{core}/**CC3**-RS_{shell} crystals. The core-shell crystals were prepared in DCM at 30°C.

Figure S9. SEM images for core crystals of **CC3**-*RS* and core-shell crystals of **CC3**-RS_{core}/**CC19**-$RS_{shell.}$ The particle size of **CC3**-RS_{core}/**CC19**-RS_{shell} is 3–5 µm as compared with a particle size of ~2 µm for the **CC3**-*RS* 'seed' crystals. The core-shell crystals were prepared in $CHCl_3$ at 50°C.

Figure S10. SEM images for core crystals of **CC19**-*RS* and core-shell crystals, **CC19**-RS_{core}/**CC3**-$RS_{shell.}$ The particle size of **CC19**-RS_{core}/**CC3**-RS_{shell} is ~3 µm, as compared with a particle size of 1–2 µm for the **CC19**-*RS* seed crystals. The core-shell crystals prepared in $CHCl_3$ at 50°C.

Figures S11. A SEM image of core-shell crystal, **CC3**-RS_{core}/**CC19**-RS_{shell}, showing evidence (terraces) of the shell layer formation.

Figures S12. Z-stack of confocal Microscopy images of **CC3**-RS_{core}/**CC19**-RS_{shell}.

Figures S13. Z-stack of confocal Microscopy images of **CC19**-RS_{core}/**CC3**-RS_{shell}.

Figure S14. PXRD patterns for **CC3**-*RS*, **CC19**-*RS*, and **CC3**-*RS*$_{core}$/**CC19**-*RS*$_{shell}$.

Table S3. The unit cell parameters for the racemic particles (**CC3**-*RS* and **CC19**-*RS*) and core-shell samples (**CC3**-*RS*$_{core}$/**CC19**-*RS*$_{shell}$ and **CC19**-*RS*$_{core}$/**CC3**-*RS*$_{shell}$)

Figure S15. (a) The carbon 1s spectra with two peaks fitted, except the sample **CC3**-*RS*$_{core}$/**CC19**-*RS*$_{shell}$ shows an extra small peak at higher binding energy. (b) The nitrogen 1s spectra with a single peak fitted. (c) The oxygen 1s spectra.

Table S4. Elemental composition results obtained by XPS.

Figure S16. Solution UV absorption spectra for **CC3**-*RS*, **CC19**-*RS*, **CC3**-*RS*$_{core}$/**CC19**-*RS*$_{shell}$ and **CC19**-*RS*$_{core}$/**CC3**-*RS*$_{shell}$.

Figure S17. (a) Solution fluorescence excitation spectra, (b) solution fluorescence emission spectra for **CC3**-*RS*, **CC19**-*RS*, **CC3**-*RS*$_{core}$/**CC19**-*RS*$_{shell}$ and **CC19**-*RS*$_{core}$/**CC3**-*RS*$_{shell}$.

Figure S18. SEM images for core crystals of **CC3**-*RS* and core-shell crystals of **CC3**-*RS*$_{core}$**/CC15***S*-**CC3***R*$_{shell}$. The particle size of **CC3**-*RS*$_{core}$**/CC15***S*-**CC3***R*$_{shell}$ was ~3 μm, as compared with a particle size of 1–2 μm for **CC3**-*RS*. The core-shell crystals were prepared in $CHCl_3$ at 50°C.

Figure S19. PXRD patterns for **CC3**-*RS*, **CC15***S*-**CC3***R*, and **CC3**-*RS*$_{core}$**/CC15***S*-**CC3***R*$_{shell}$.

Figure S20. Contact angle measurement for (a) **CC3**-*RS*, (b) **CC3**-*RS*$_{core}$/**CC19**-*RS*$_{shell}$, (c) **CC19**-*RS*, (d) **CC19**-*RS*$_{core}$/**CC3**-*RS*$_{shell}$, (e) **CC3**-*R*/**CC15**-*S* and (f) **CC3**-*RS*$_{core}$/**CC15***S*-**CC3***R*$_{shell}$.

Figure S21. Gas sorption isotherms at 77 K for N_2 (a) and H_2 (b) for **CC3**-*RS*, **CC19**-*RS*, **CC3**-*RS*$_{core}$/**CC19**-*RS*$_{shell}$ and **CC19**-*RS*$_{core}$/**CC3**-*RS*$_{shell}$.

Figure S22. Pressure-dependent IAST selectivity of CO_2 over CH_4, as determined for equimolar mixtures using experimental single-component isotherms at 273 K: (a) **CC19-***RS*$_{core}$/**CC3-***RS*$_{shell}$; (b) **CC3-***RS*$_{core}$/**CC19-***RS*$_{shell}$.

Table S5. Gas sorption data of **CC3**-*RS*, **CC19**-*RS*, **CC3**-*RS*$_{core}$/**CC19**-*RS*$_{shell}$ and **CC19**-*RS*$_{core}$/**CC3**-*RS*$_{shell}$.

Figure S23. (a) STEM image for **CC3**-*RS*$_{core}$/**CC19**-*RS*$_{shell}$ without gold coating, (b) TEM image for **CC3**-*RS*$_{core}$/**CC19**-*RS*$_{shell}$, (c) STEM image for **CC19**-*RS*$_{core}$/**CC3**-*RS*$_{shell}$, (d) TEM image for **CC19**-*RS*$_{core}$/**CC3**-*RS*$_{shell}$.

Figure S24. Confocal microscopy images for dye uptake in the core-shell cage crystals.

Figure S25. SEM images showing examples of damaged core-shell crystals in the sample.

Methods

Materials. 1,3,5-Triformylbenzene was purchased from Manchester Organics, (1*R*,2*R*)-(-)-1,2-cyclohexanediamine, (1*S*,2*S*)-(-)-1,2-cyclohexanediamine and 1,3,5-triacetylbenzene were purchased from TCI-UK, and 2-hydroxy-1,3,5-benzenetricarbaldehyde was purchased from Key Organics Ltd. All other chemicals were purchased from Fisher and used as received.

Cage synthesis: CC3, CC15 and CC19 were all reported previously.[1]

CC3 synthesis: **CC3**-*R* was synthesized by the condensation reaction of 1, 3, 5-triformylbenzene (TFB) and (1*R*, 2*R*)-(-)-1,2-diaminocyclohexane (*R*, *R*-CHDA) in DCM at room temperature. TFB (400 mg, 2.47 mmol) was dissolved in DCM (20 ml) and added to a solution of *R*,*R*-CHDA (422 mg, 3.70mmol) in DCM (20 ml). After 5–7 days, crystals were formed and isolated to obtain white powders with a yield of 70%. The synthesis of **CC3**-*S* was identical to that of **CC3**-*R*, apart from the use of the (1*S*, 2*S*)-(-)-1,2-diaminocyclohexane (*S*,*S*-CHDA).

CC19 synthesis: for **CC19**-*R*, DCM (50 mL) was added to 2-hydroxy-1,3,5-benzenetricarbaldehyde (50 mg, 0.31 mmol) in a round bottom flask at room temperature. After 5 minutes, a solution of *R*,*R*-CHDA (34 mg, 0.47 mmol) in MeOH (5 mL) was added. The resulting mixture was left covered for 3–5 days with stirring. A yellow powders, **CC19**-*R*, was obtained by slow evaporation with a yield of 78 %. Crystals were obtained by dissolving the **CC19**-*R* powder in $CHCl_3$ solution and the crystals can be isolated over several days with slow evaporation at room temperature. The opposite cage enantiomer was formed from *S*,*S*-CHDA.

CC15-*S* synthesis: a solution of *S*,*S*-CHDA (4.28 g, 37.5 mmol) in DCM (50 mL) was added to 1,3,5-triacetylbenzene (TAB) (5.10 g, 25.0 mmol) and molecular sieves (1 g, 3 Å), giving a yellow suspension. The flask was equipped with reverse Dean-Stark apparatus, which was charged with DCM, and the solution was heated to 45 °C with stirring for 24 h. Approximately 0.7 mL of water was collected in the Dean-Stark apparatus. The reaction was then cooled to room temperature and filtered by gravity to remove the molecular sieves. The resulting pale yellow solution was evaporated to yield a pale yellow solid (92% crude yield), which was recrystallized from DCM/hexane (1:1) to yield white needle-like crystals.

Cage core-shell synthesis: In the previous study, Hasell *et al*. described a method for fabrication of racemic cage nanoparticles using chiral recognition when mixing the cage molecule with an opposite chirality.[2] Core-shell morphologies were achieved using an adapted version of this procedure via the growth of the racemic (or quasiracemic) cage shell in the presence of the core crystals, which act as seeds. All synthetic details are described in the main manuscript.

CC3-*RS*$_{core}$/**CC19**-*RS*$_{shell}$ core-shell crystals with a particle size of < 1 µm: 1 equivalent **CC3**-*R* DCM solution (2 mg/ml, 1 ml) was prepared, and 1 equivalent of **CC3**-*S* DCM solution (2 mg/ml, 1 ml) was added into the **CC3**-*R* solution using a syringe pump with a rate of mixing of 5 ml/h. The mixed the solution was left over 4 h to form the racemic core crystals. Once the core crystals were formed, 1 equivalent **CC19**-*R* DCM solution (2.1 mg/ml, 1 ml) and 1 equivalent of **CC19**-*S* (2.1 mg/ml, 1ml) were added using a syringe pump to form the racemic shell. The preparation was carried out at 30 °C. The as-prepared solution was slowly evaporated to obtain the core-shell crystals as a powder.

CC3-*RS*$_{core}$/**CC19**-*RS*$_{shell}$ core-shell crystals with a particle size of 3–4 µm: 1 equivalent **CC3**-*R* $CHCl_3$ solution (8 mg/ml, 1 ml) was prepared, and 1 equivalent of **CC3**-*S* $CHCl_3$ solution (8 mg/ml, 1 ml) was added into the **CC3**-*R* solution using a syringe pump with a rate of mixing of 5 ml/h. The mixed solution was left over 4 h to form the racemic core crystals. Once the core crystals were formed, 1 equivalent **CC19**-*R* $CHCl_3$ solution (8.5 mg/ml, 1 ml) and 1 equivalent of **CC19**-*S* (8.5 mg/ml, 1ml) were added using a syringe pump to form the racemic shell. The preparation was carried out at 50 °C, which led to larger particle sizes (c.f., preparation above at 30 °C). The as-prepared solution was slowly evaporated to obtain the core-shell crystals as a powder. The synthesis of **CC3**-*RS*$_{core}$/**CC19**-*RS*$_{shell}$ core-shell crystals with a particle size of 5–6 µm for confocal microscopy studies was identical to that of core-shell crystals with particle size of 3-4 µm, apart from the preparation carried out in $CHCl_3$ at 60 °C.

A similar synthetic procedure was applied for the inverse **CC19**-*RS*$_{core}$/**CC3**-*RS*$_{shell}$ core-shell crystals; the components were simply added in the reverse order.

CC3-*RS*$_{core}$/**CC15***S*-**CC3***R*$_{shell}$ core-shell crystals with a particle size of 3 µm: 1 equivalent **CC3**-*R* DCM solution (2 mg/ml, 1 ml) was prepared, and 1 equivalent of **CC3**-*S* DCM solution (2 mg/ml, 1 ml) was added into the **CC3**-*R* solution using a syringe pump with a rate of mixing of 5 ml/h. The mixed the solution was left over 4 h to form the quasiracemic core crystals. Once the core crystals were formed, 1 equivalent **CC3**-*R* DCM solution (2 mg/ml, 1 ml) and 1 equivalent of **CC15**-*S* (2.3 mg/ml, 1ml) were added using a syringe pump to form shell-cocrystals The preparation was carried out at 30 °C. The as-prepared solution was slowly evaporated to obtain the core-shell crystals as a powder.

Characterization
NMR
Solution ^{1}H NMR spectra were recorded in deuterated chloroform at 400.13 MHz using a Bruker Avance 400 NMR spectrometer.

Scanning Electron Microscopy
Imaging of the crystal morphology was achieved using a Hitachi S-4800 cold field emission scanning electron microscope (FE-SEM) operating in both scanning and transmission modes. Scanning-mode samples were prepared by depositing dry crystals on 15 mm Hitachi M4 aluminum stubs using an adhesive high-purity carbon tab before coating with a 2 nm layer of gold using an Emitech K550X automated sputter coater. Imaging was conducted at a working distance of 8 mm and a working voltage of 3 kV using a mix of upper and lower secondary electron detectors. Transmission-mode samples were prepared by dispersing the cage particles in a methanol suspension and depositing onto carbon-coated copper grids (300 mesh), imaging at 30 kV working voltage and 7 mm distance.

Dynamic Light Scattering
Analysis was performed directly on the mixed cage solutions in DCM or $CHCl_3$ using quartz vials and a Malvern Instruments Zetasizer nano series.

Powder X-ray Diffraction
High-resolution synchrotron PXRD data were collected on the I11 beamline at Diamond Light Source (λ = 0.825865 Å) in transmission geometry using the Mythen II positive sensitive detector. Samples were contained in 0.7 mm diameter borosilicate glass capillaries and spun to improve powder averaging. Analysis of the XRD profiles was performed using *TOPAS-Academic*.[3]

Gas Sorption Analysis
Surface areas were measured by nitrogen adsorption and desorption at 77.3 K. Powder samples were degassed offline at 100 °C for 15 h under dynamic vacuum before analysis, followed by degassing on the analysis port under vacuum, also at 100 °C. Isotherms were measured using a Micromeritics 2020, 2420 or 2050 volumetric adsorption analyzer.

Confocal Microscopy

Core-shell crystals were imaged with confocal microscopy using a Zeiss LSM510 scan head on a Zeiss Observer Z1 (Zeiss, Jena, Germany) with a Plan Apochromat 63x/NA =1.4. The excitation source used was an argon ion laser at 488 nm. Images were collected and analyzed using the Zeiss AIM software (Zeiss, Jena, Germany).

Imaris (version 8.2.0, Oxford Instruments/Bitplane, Zurich, Switzerland) was used to create the 3D model of the core shell particle. The GFP channel was segmented into 3D volumes by absolute intensity using an automatically selected intensity threshold.

X-Ray Photoelectron Spectroscopy (XPS) Surface Analysis

XPS measurements were conducted using a SPECS monochromatic Al Kα (1486.6 eV) X-ray source typically operating at 200 W, together with a PSP Vacuum Technology electron-energy analyzer operating with a typical constant pass energy of 10 eV. Further details, including spectrometer calibration, can be found in the reference. [4]

(a)

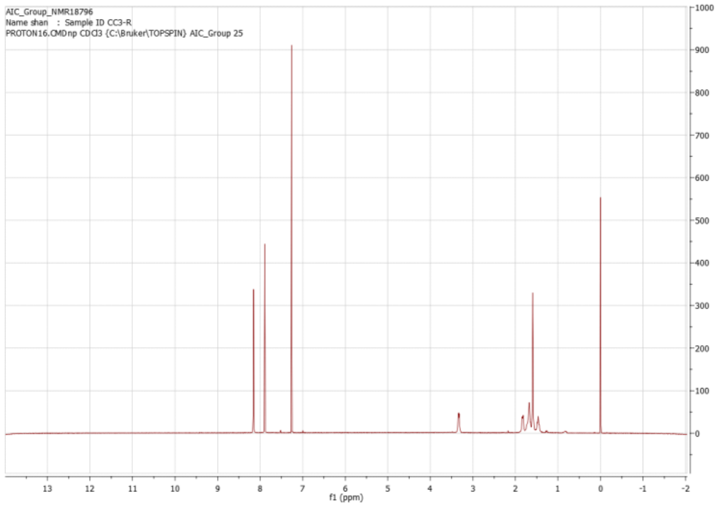

(b)

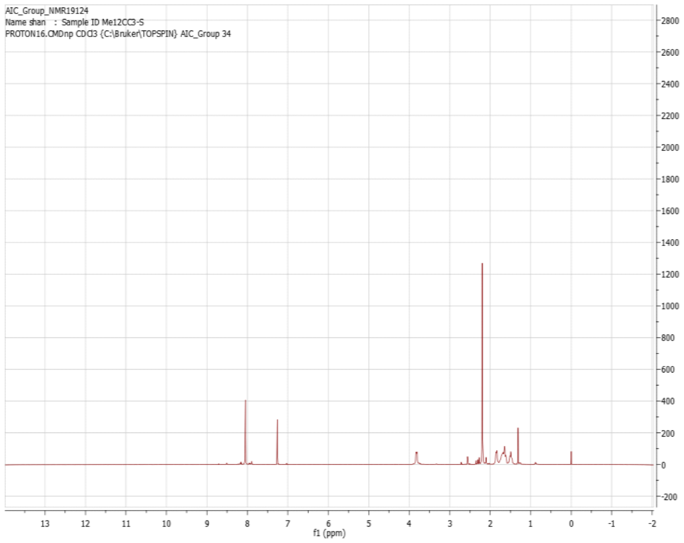

(c)

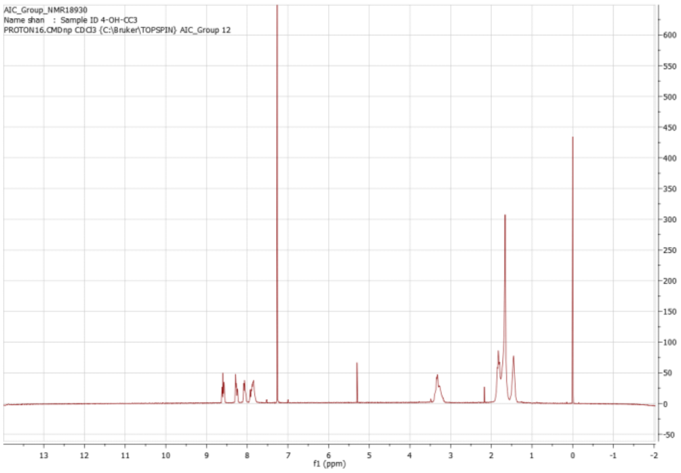

Figure S1. ^{1}H NMR spectrum ($CDCl_3$) of (a) **CC3**-*R*, (b) **CC15**-*R* and (c) **CC19**-*R*.

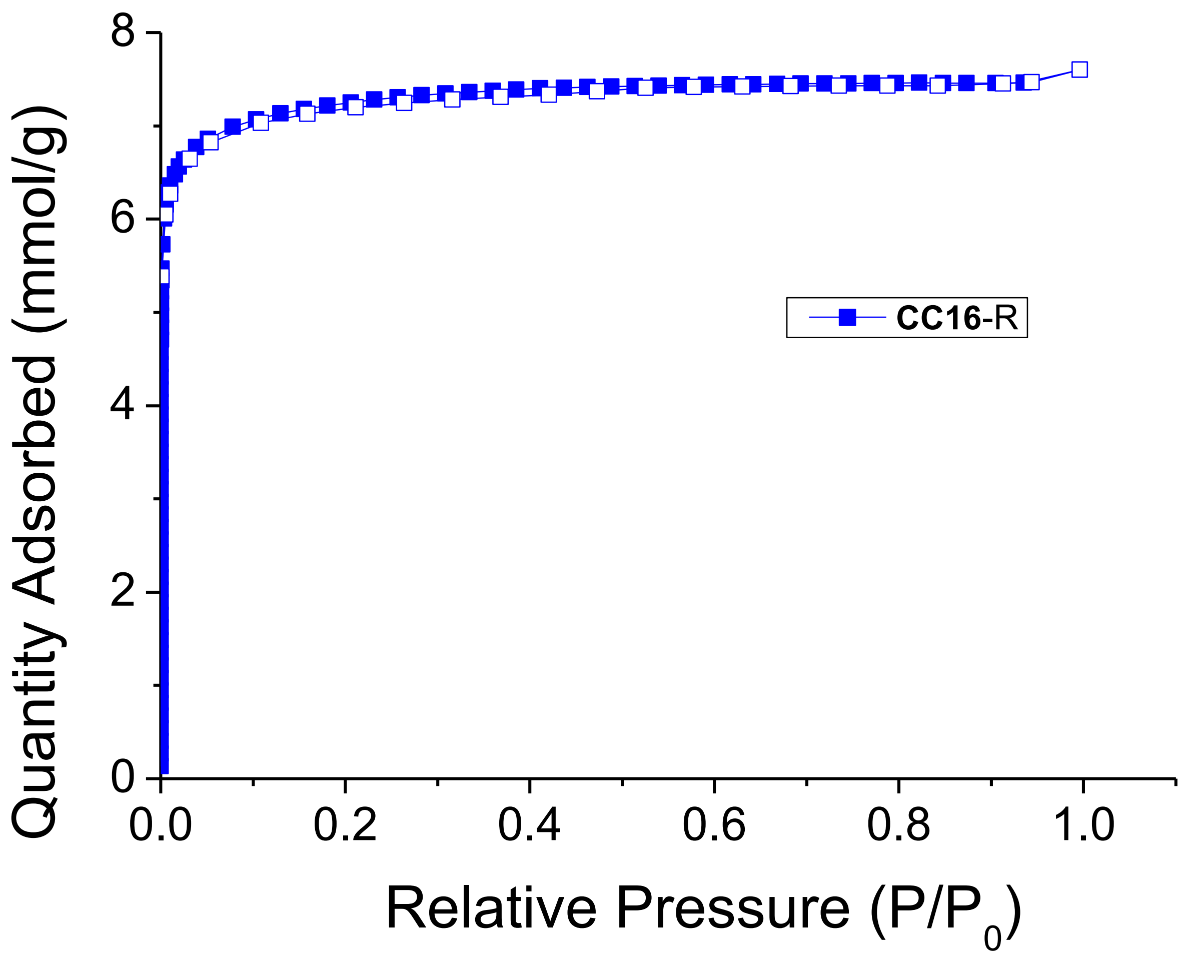

Figure S2. Gas sorption isotherm for N_2 at 77 K for **CC19**-*R*. Filled and open symbols represent adsorption and desorption isotherms respectively.

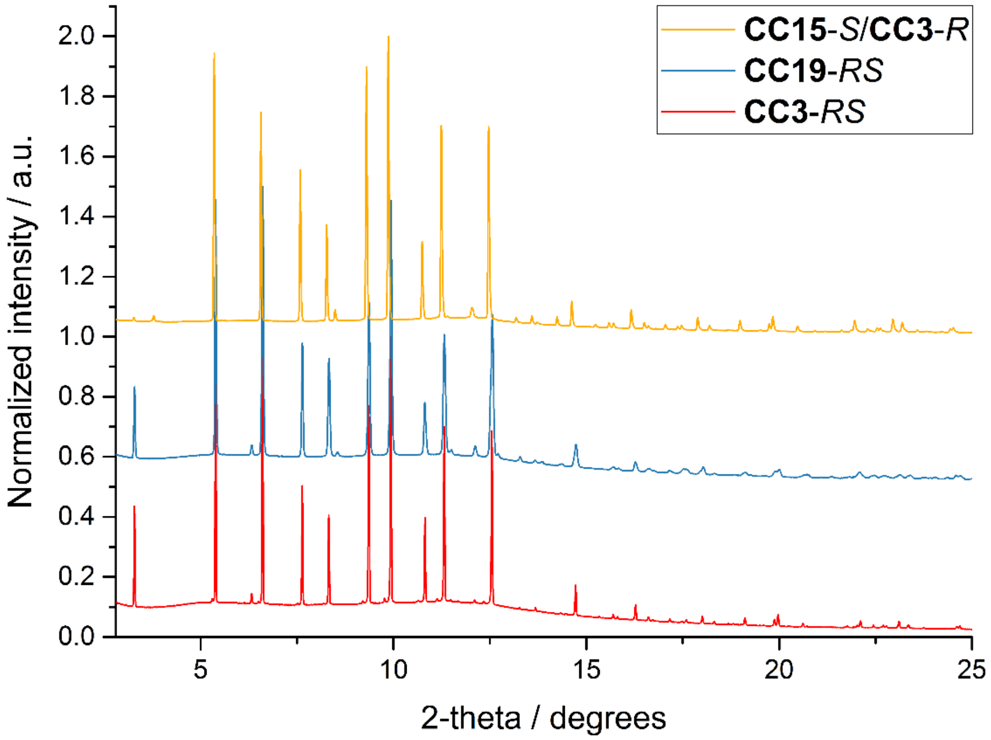

Figure S3. PXRD patterns for racemic **CC3**-*RS*, **CC19**-*RS*, and quasi-racemic **CC3**-*R*/**CC15**-*S*.

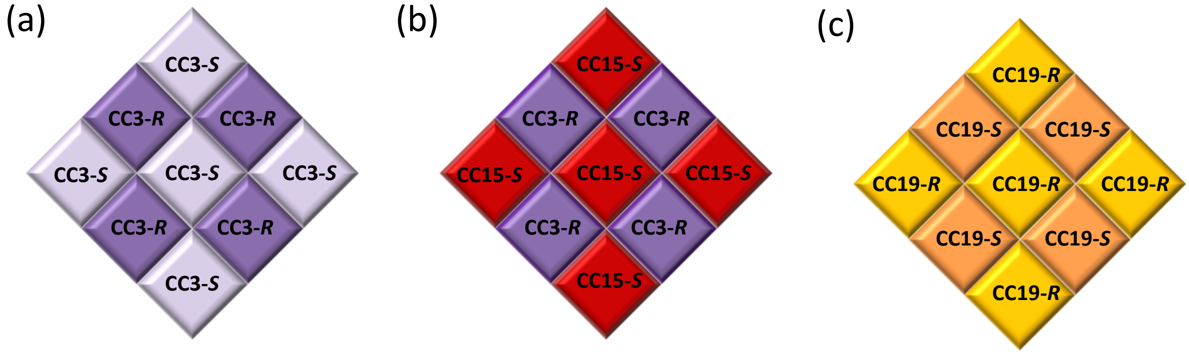

Figure S4. Scheme showing the cage packing of (a) racemic **CC3**-*RS*, (b) quasi-racemic **CC3**-*R*/**CC15**-*S* and (c) racemic **CC19**-*RS*.

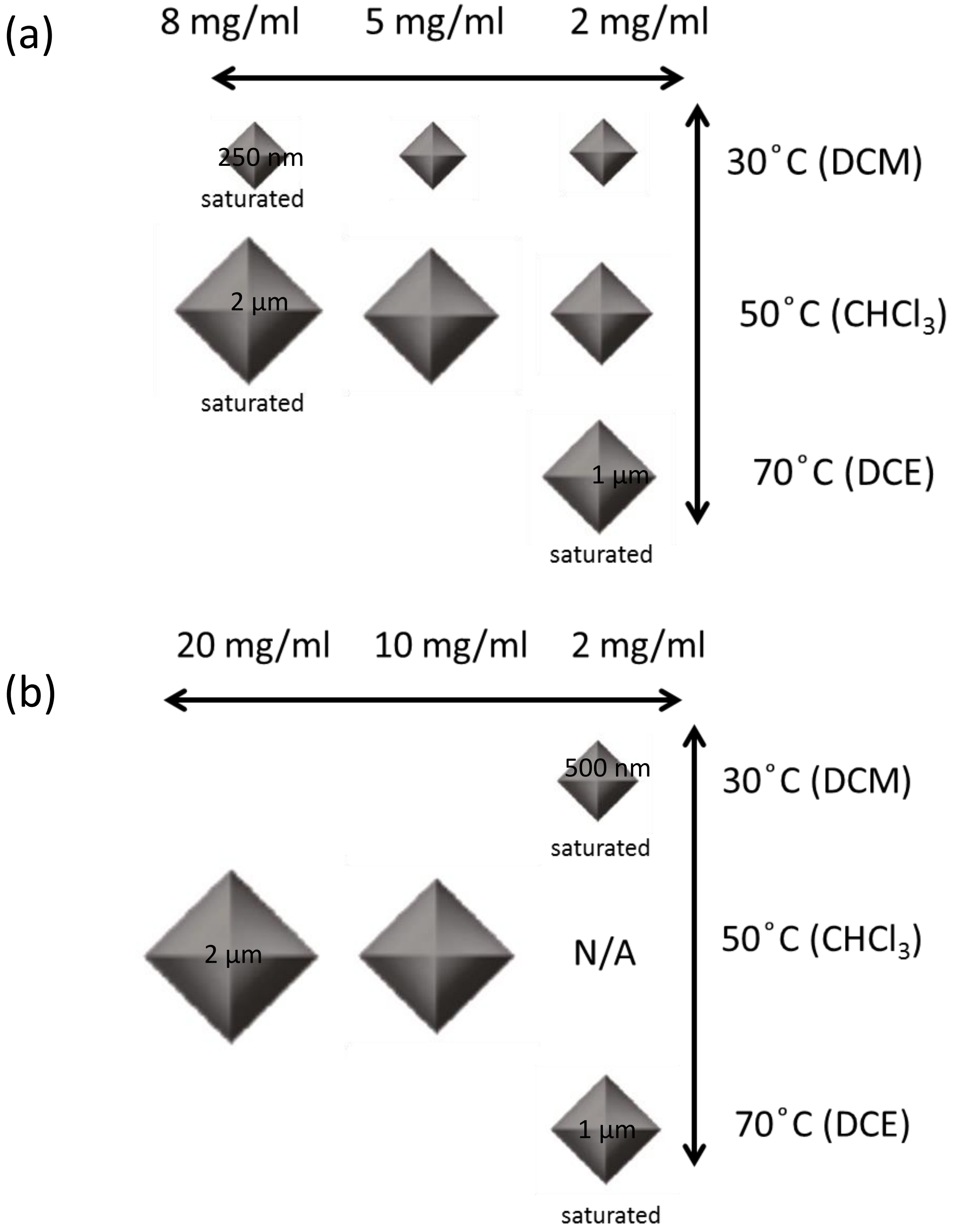

Figure S5. (a) Particle size distributions of **CC3**-*RS* prepared at various mixing solution concentrations and different solvent systems. (b) Particle size distributions of **CC19**-*RS*. The temperature was maintained at 30 °C for DCM, 50 °C for CHCl3, and 70 °C for 1, 2-dichloroethane (DCE) solvent.

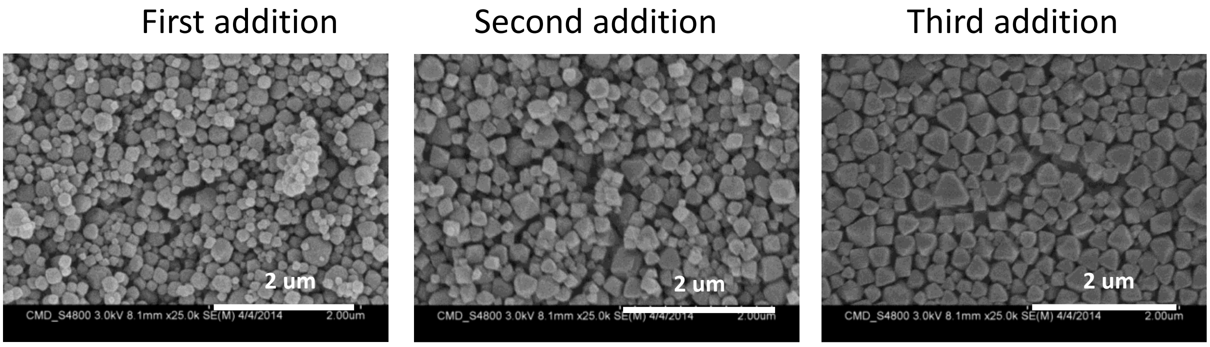

Figure S6. SEM images for racemic **CC3** particles prepared by the sequential addition of **CC3**-*R* and **CC3**-*S* solutions**.** (a) SEM image for the cage particles from the first addition; (b) second addition; (c) third addition.

Table S1. Particle sizes for the racemic **CC3**-*RS* as prepared by the sequential addition method.

	Z-AVE (d, nm)
**CC3**-*RS* first addition	193.4
**CC3**-*RS* second addition	239.5
**CC3**-*RS* third addition	310.8

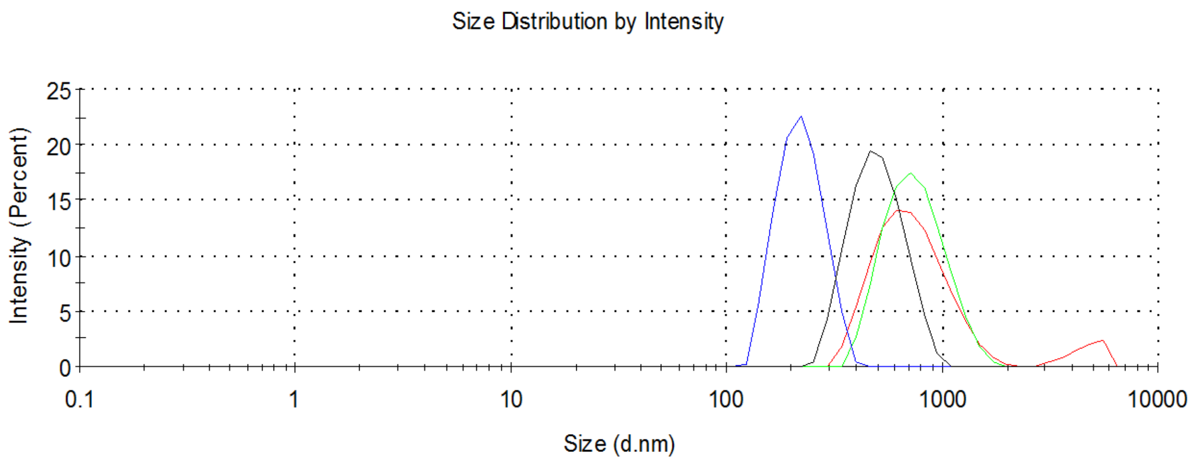

Figure S7. Particle size distributions from dynamic light scattering (DLS) for **CC3**-*RS* (blue curve), **CC19**-*RS* (black curve), **CC3**-*RS*$_{core}$/**CC19**-*RS*$_{shell}$ (red curve) and **CC19**-*RS*$_{core}$/**CC3**-*RS*$_{shell}$ (green curve). The samples were prepared in DCM solution at 30 ˚C.

Table S2. Particle sizes calculated from DLS.

	Z-AVE (d, nm)	PDI
**CC3**-*RS*	212	0.048
**CC19**-*RS*	474	0.107
**CC3**-*RS*$_{core}$/**CC19**-*RS*$_{shell}$	744	0.227
**CC19**-*RS*$_{core}$/**CC3**-*RS*$_{shell}$	721	0.128

CC3-*RS*$_{core}$/**CC19**-*RS*$_{shell}$

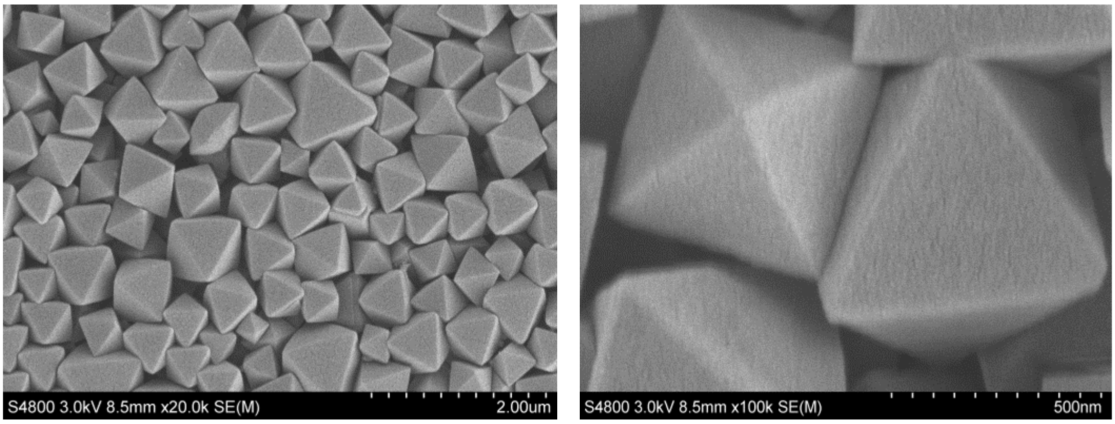

CC19-*RS*$_{core}$/**CC3**-*RS*$_{shell}$

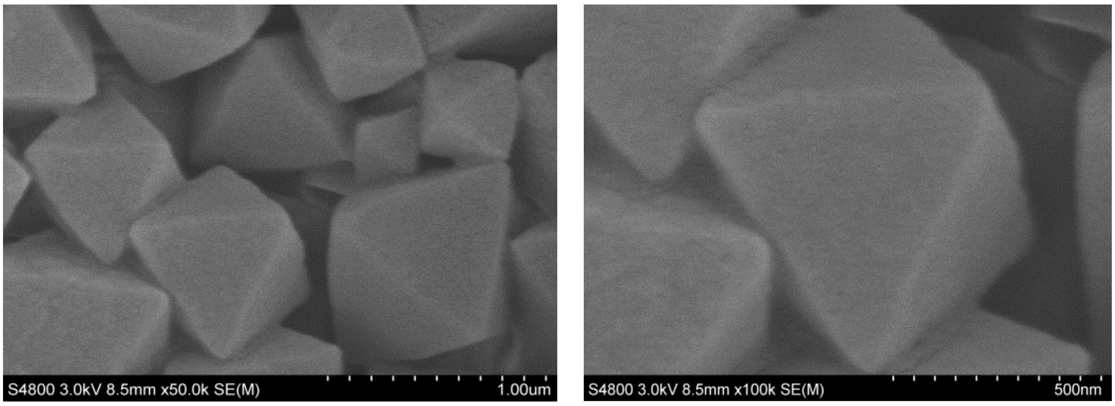

Figure S8. SEM images for **CC3**-*RS*$_{core}$/**CC19**-*RS*$_{shell}$ and **CC19**-*RS*$_{core}$/**CC3**-*RS*$_{shell}$ crystals. The core-shell crystals were prepared in DCM at 30°C.

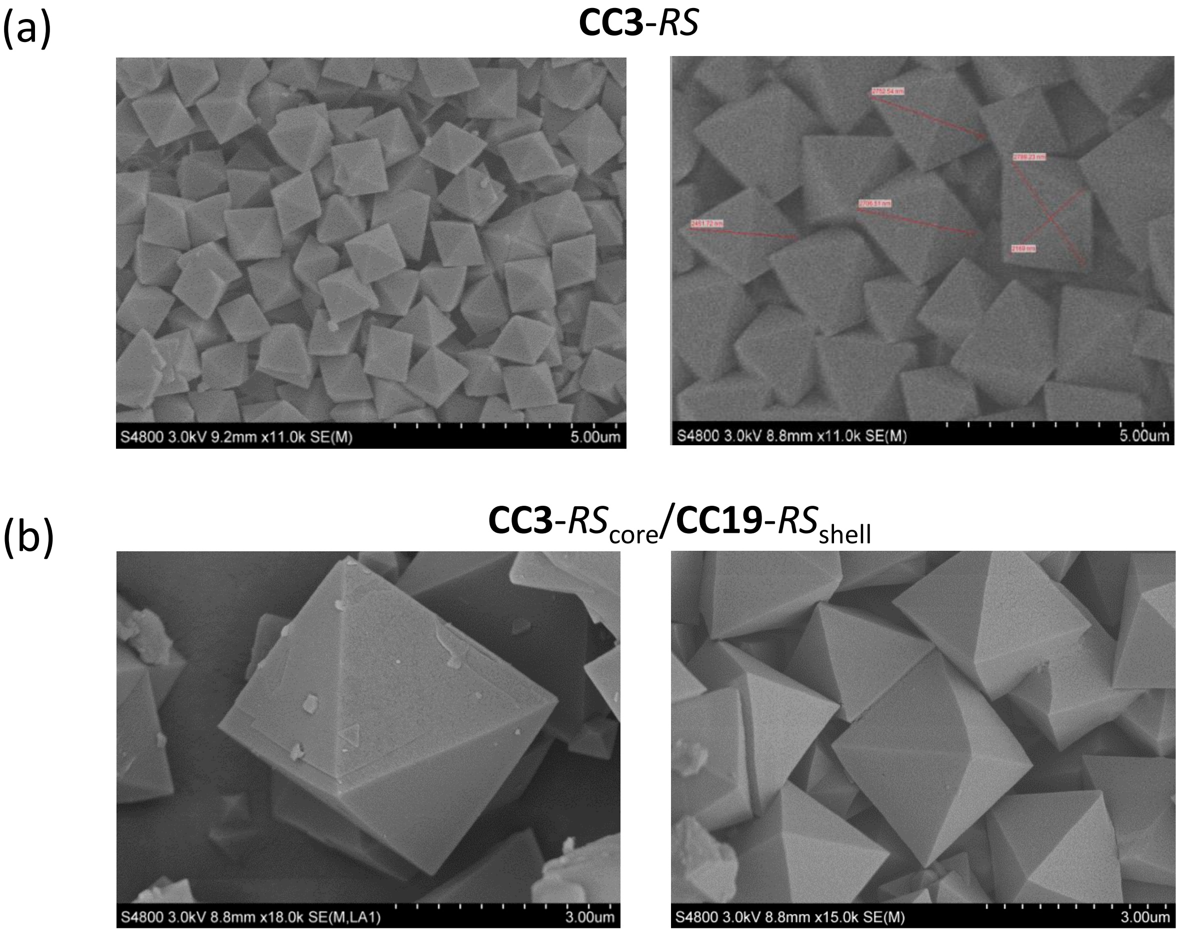

Figure S9. SEM images for core crystals of **CC3**-*RS* and core-shell crystals of **CC3**-*RS*$_{core}$/**CC19**-*RS*$_{shell.}$ The particle size of **CC3**-*RS*$_{core}$/**CC19**-*RS*$_{shell}$ is 3–5 µm as compared with a particle size of ~2 µm for the **CC3**-*RS* ‘seed’ crystals. The core-shell crystals were prepared in $CHCl_3$ at 50°C.

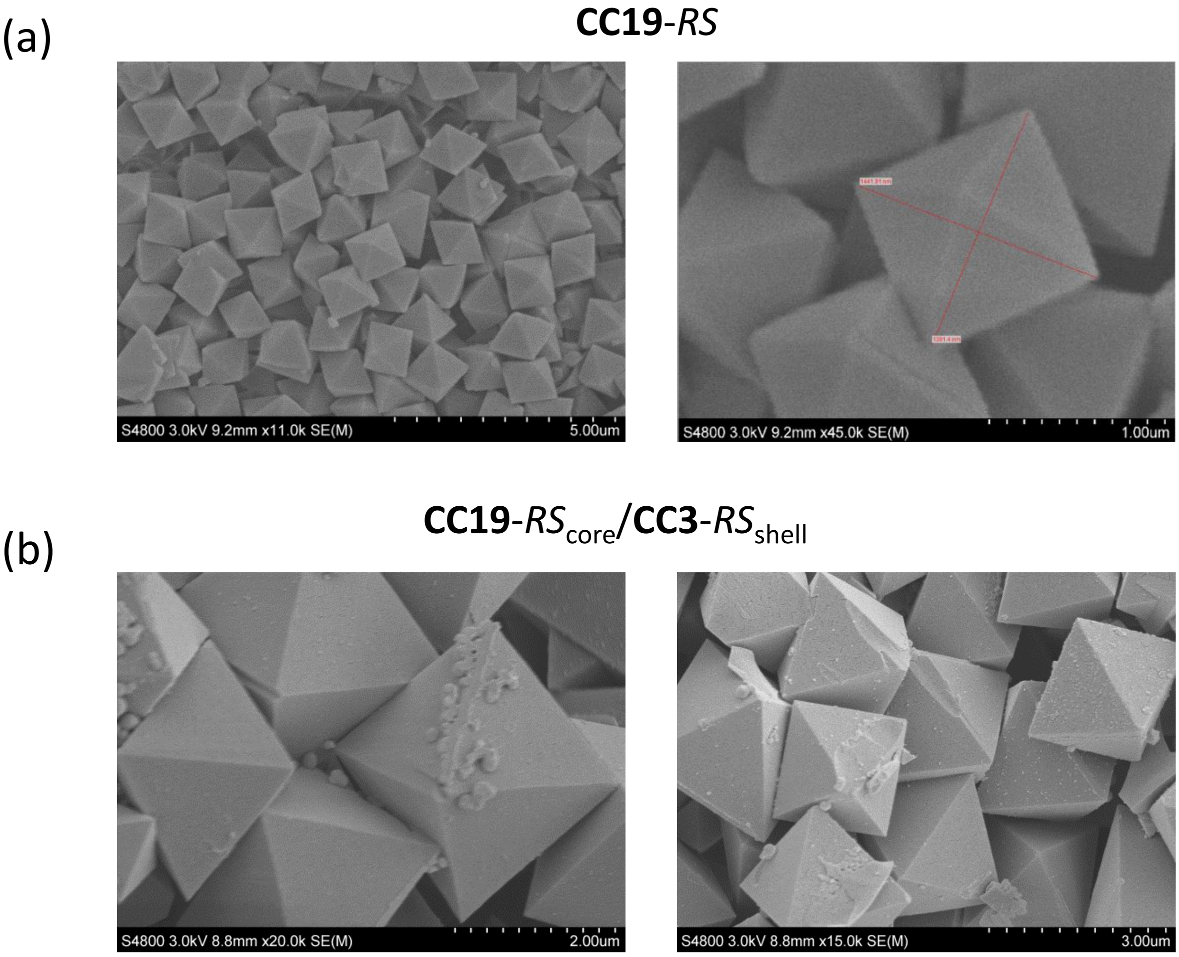

Figure S10. SEM images for core crystals of **CC19**-*RS* and core-shell crystals, **CC19**-*RS*$_{core}$/**CC3**-*RS*$_{shell.}$ The particle size of **CC19**-*RS*$_{core}$/**CC3**-*RS*$_{shell}$ is ~3 µm, as compared with a particle size of 1–2 µm for the **CC19**-*RS* seed crystals. The core-shell crystals prepared in $CHCl_3$ at 50°C.

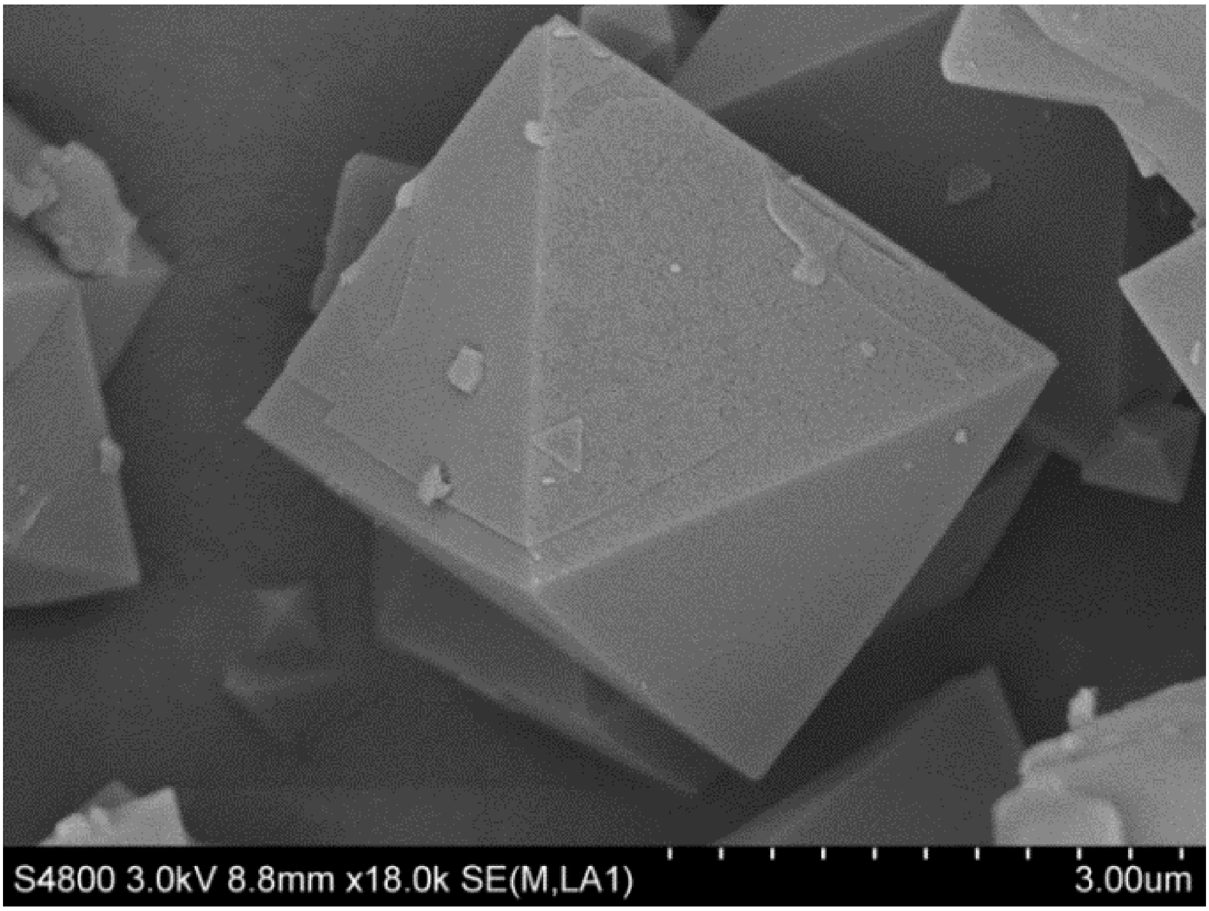

Figures S11. A SEM image of core-shell crystal, **CC3**-*RS*$_{core}$/**CC19**-*RS*$_{shell}$, showing evidence (terraces) of the shell layer formation.

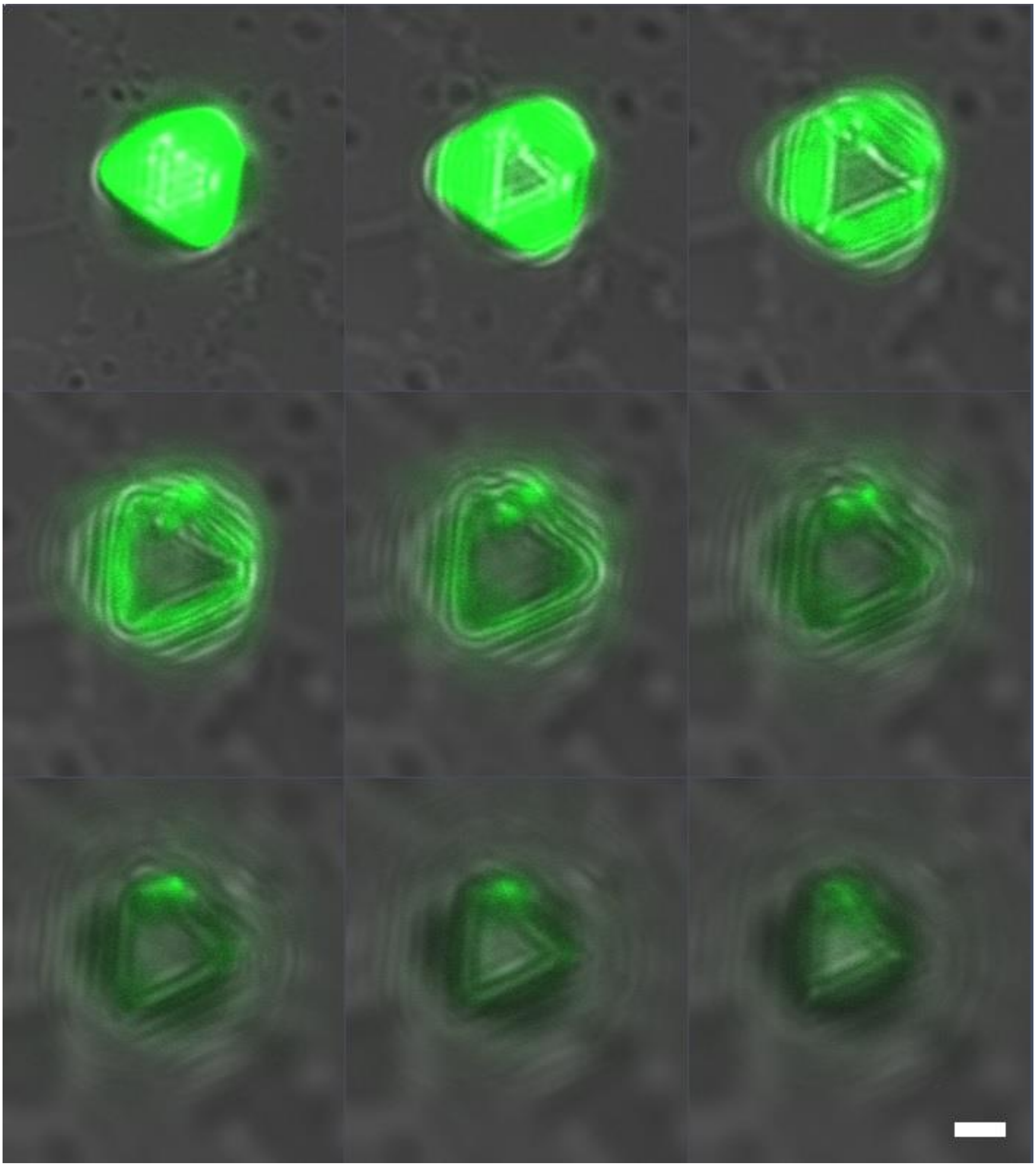

Figures S12. Z-stack of confocal Microscopy images of **CC3**-*RS*$_{core}$/**CC19**-*RS*$_{shell}$. The images were captured from the top surface to the bottom of crystal. Scale bar, 1 micron.

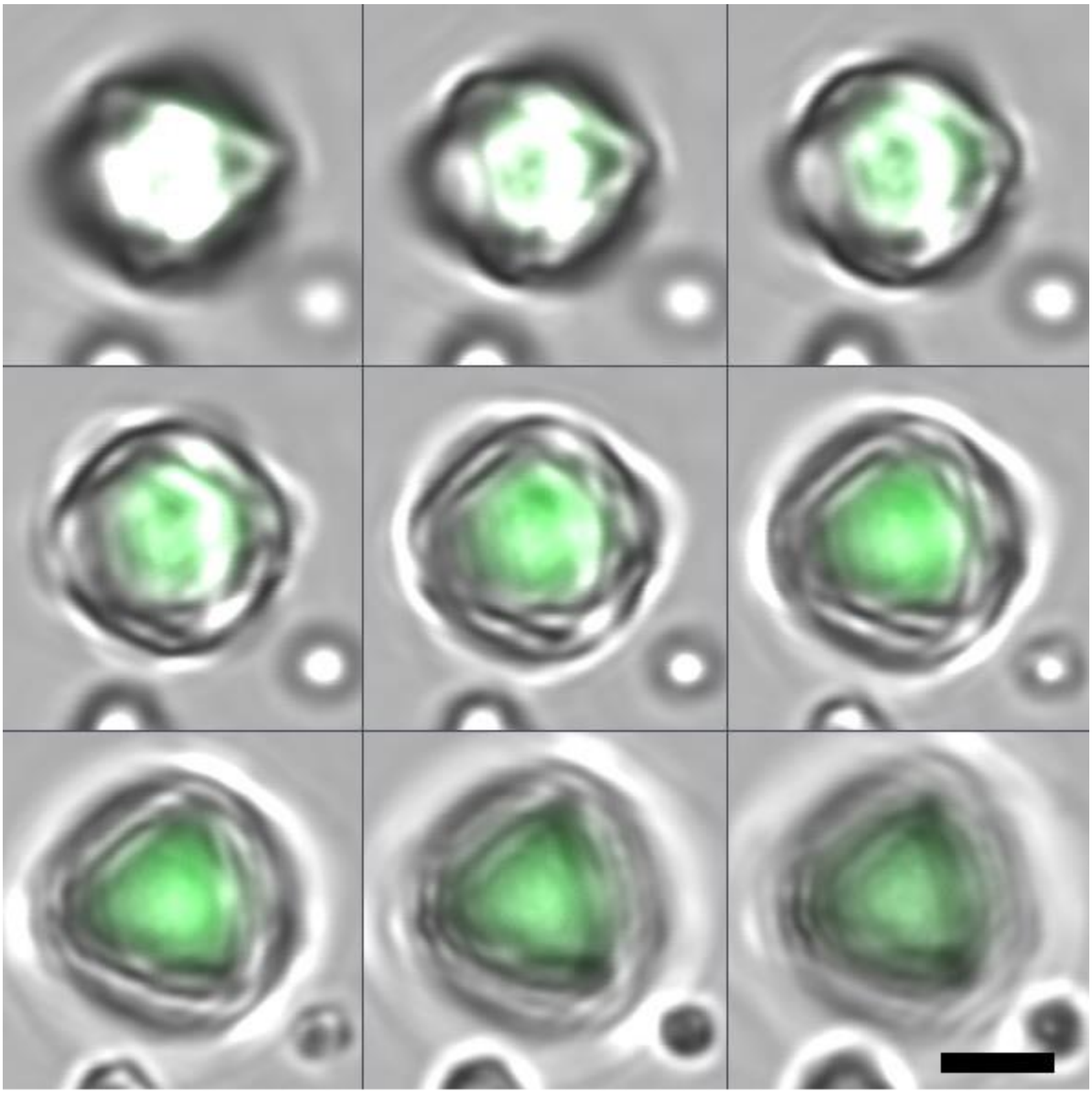

Figures S13. Z-stack of confocal Microscopy images of **CC19**-RS_{core}/**CC3**-RS_{shell}. The images were captured from the top surface to the bottom of crystal. Scale bar, 2 microns.

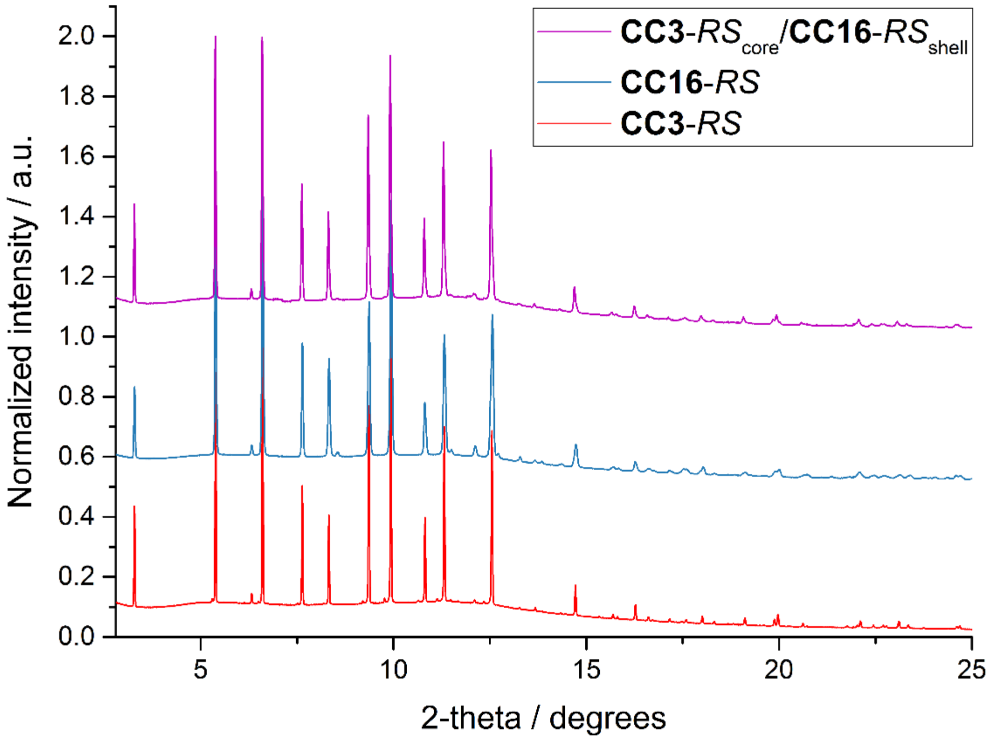

Figure S14. PXRD patterns for **CC3**-*RS*, **CC19**-*RS*, and **CC3**-RS_{core}/**CC19**-RS_{shell}.

Table S3. The unit cell parameters for the racemic particles (**CC3**-*RS* and **CC19**-*RS*) and core-shell samples (**CC3**-RS_{core}/**CC19**-RS_{shell} and **CC19**-RS_{core}/**CC3**-RS_{shell})

	**CC3**-*RS*	**CC19**-*RS*	**CC3**-RS_{core}/ **CC19**-RS_{shell}	**CC19**-RS_{core}/ **CC3**-RS_{shell}
a / Å	24.7069(1)	24.6914(3)	24.7462(3)	24.7708(2)
V / Å^3	15081.9(2)	15053.4(5)	15153.9(6)	15199.2(4)
R_{wp} / %	1.44	2.79	3.15	1.25
[illegible]^2	4.11	8.72	9.36	4.43

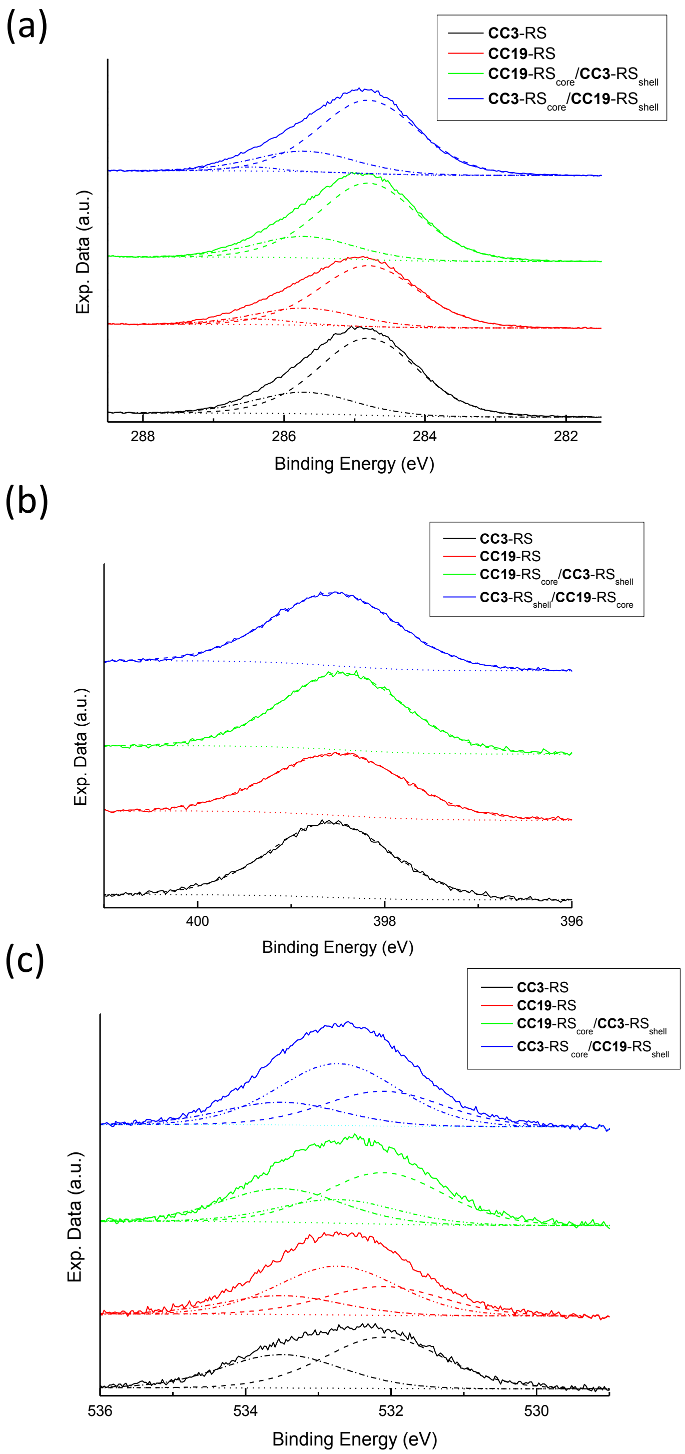

Figure S15. (a) The carbon 1s spectra with two peaks fitted, except the sample **CC3**-*RS*core/**CC19**-*RS*shell shows an extra small peak at higher binding energy. (b) The nitrogen 1s spectra with a single peak fitted. (c) The oxygen 1s spectra.

Table S4. Element composition results obtained from XPS. The oxygen composition in **CC3**-*RS*$_{core}$/**CC19**-*RS*$_{shell}$ is 5.22%, compared with 1.96% oxygen composition in **CC19**-*RS*$_{core}$/**CC3**-*RS*$_{shell}$.

	**C**	**N**	**O** (OH)
**CC3**-*RS*	84.12	8.71	0
**CC19**-*RS*	81.38	9.12	4.81
**CC3**-*RS*$_{core}$/**CC19**-*RS*$_{shell}$	82.09	8.88	1.96
**CC19**-*RS*$_{core}$/**CC3**-*RS*$_{shell}$	81.26	8.68	5.22

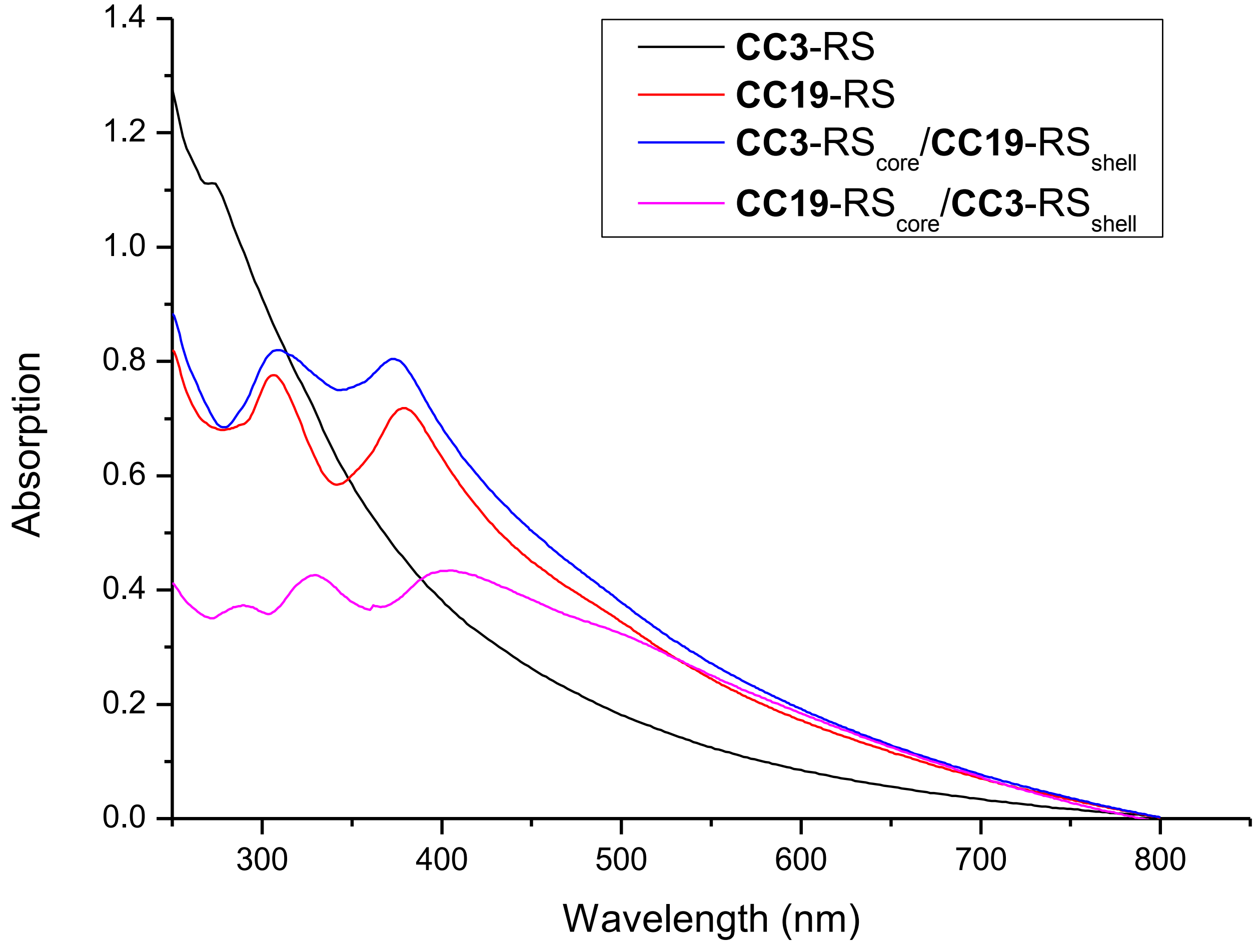

Figure S16. Solution UV absorption spectra for **CC3**-*RS*, **CC19**-*RS*, **CC3**-*RS*$_{core}$/**CC19**-*RS*$_{shell}$ and **CC19**-*RS*$_{core}$/**CC3**-*RS*$_{shell}$.

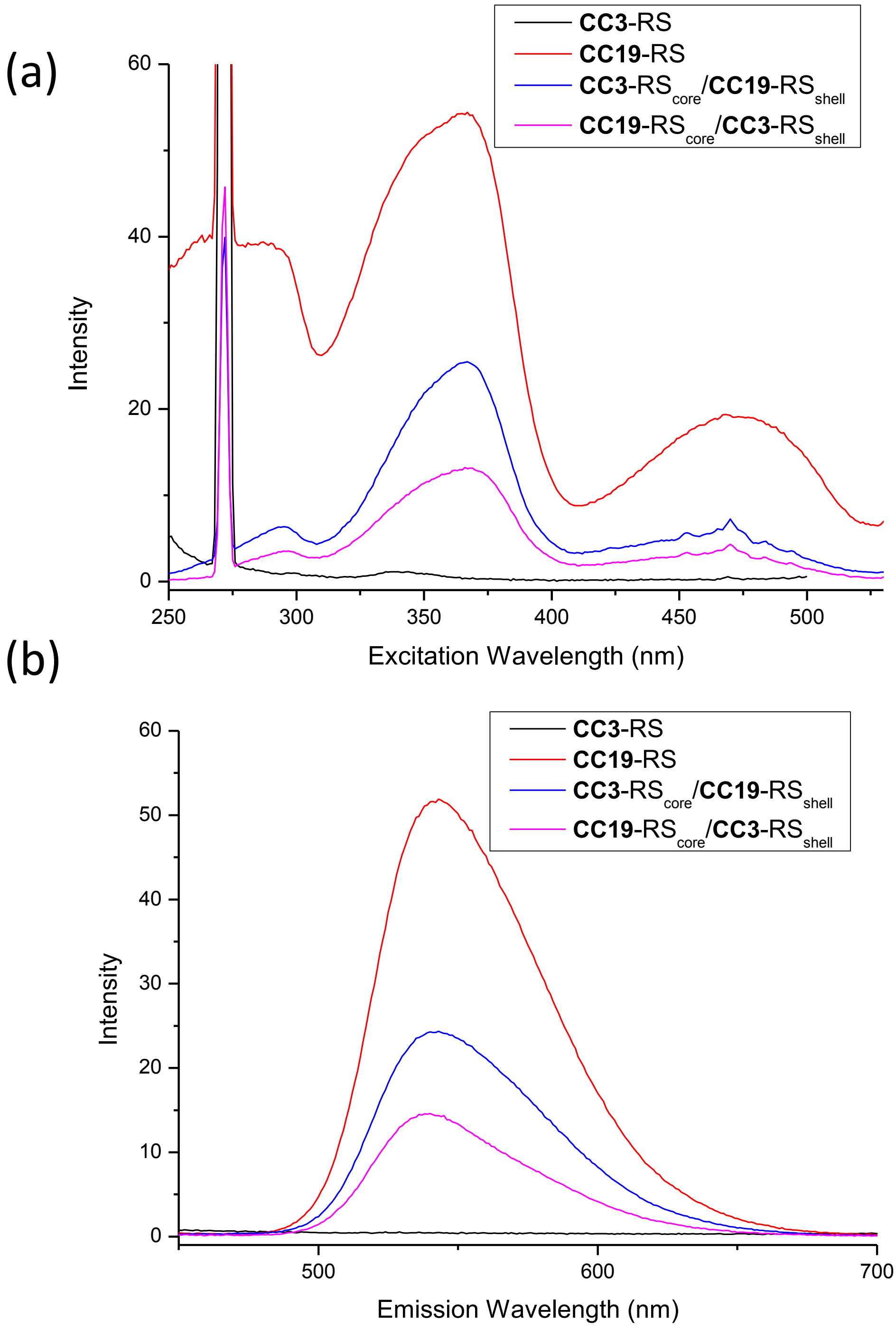

Figure S17. (a) Solution fluorescence excitation spectra, (b) solution fluorescence emission spectra for **CC3**-*RS*, **CC19**-*RS*, **CC3**-*RS*$_{core}$/**CC19**-*RS*$_{shell}$ and **CC19**-*RS*$_{core}$/**CC3**-*RS*$_{shell}$.

(a) **CC3**-*RS*

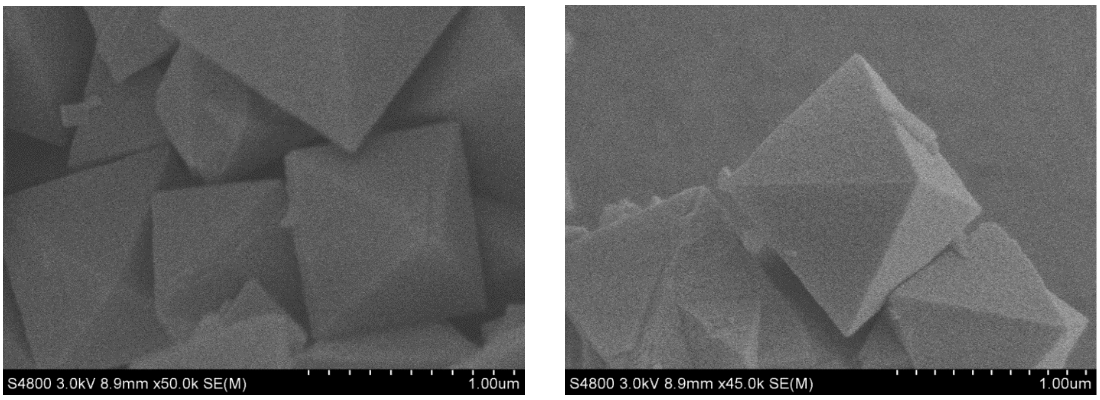

(b) **CC3**-RS_{core}/**CC15***S*-**CC3**R_{shell}

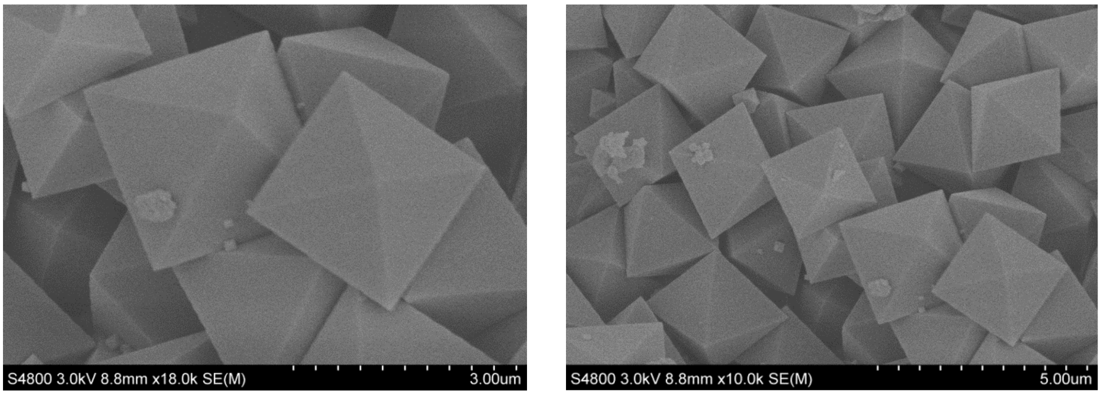

Figure S18. SEM images for core crystals of **CC3**-*RS* and core-shell crystals of **CC3**-RS_{core}/**CC15***S*-**CC3**$R_{shell.}$ The particle size of **CC3**-RS_{core}/**CC15***S*-**CC3**R_{shell} was ~3 µm, as compared with a particle size of 1–2 µm for **CC3**-*RS*. The core-shell crystals were prepared in $CHCl_3$ at 50°C.

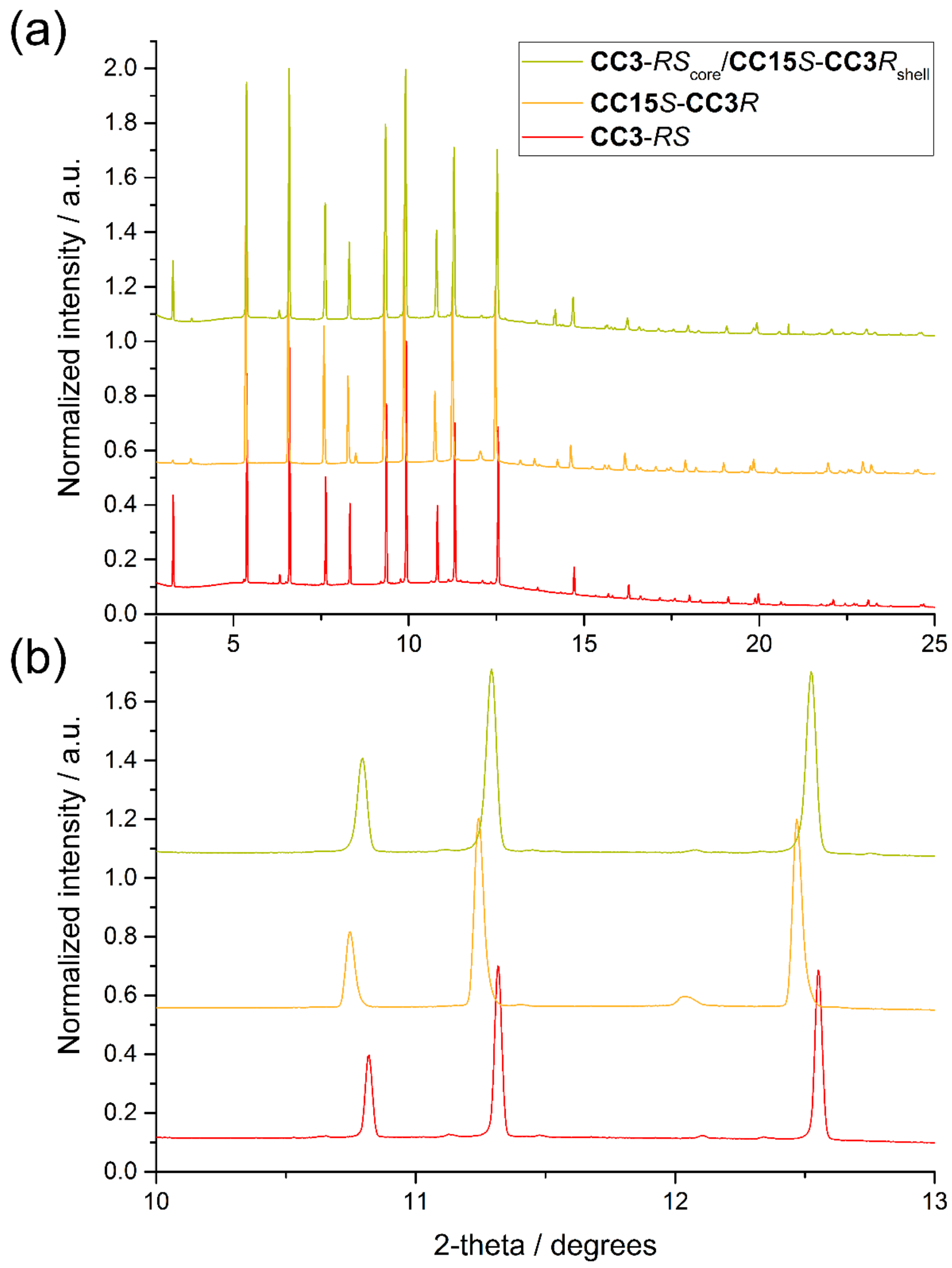

Figure S19. (a) PXRD patterns for **CC3**-*RS*, **CC15***S*-**CC3***R*, and **CC3**-*RS*$_{core}$**/CC15***S*-**CC3***R*$_{shell}$ and (b) shown at an expanded scale.

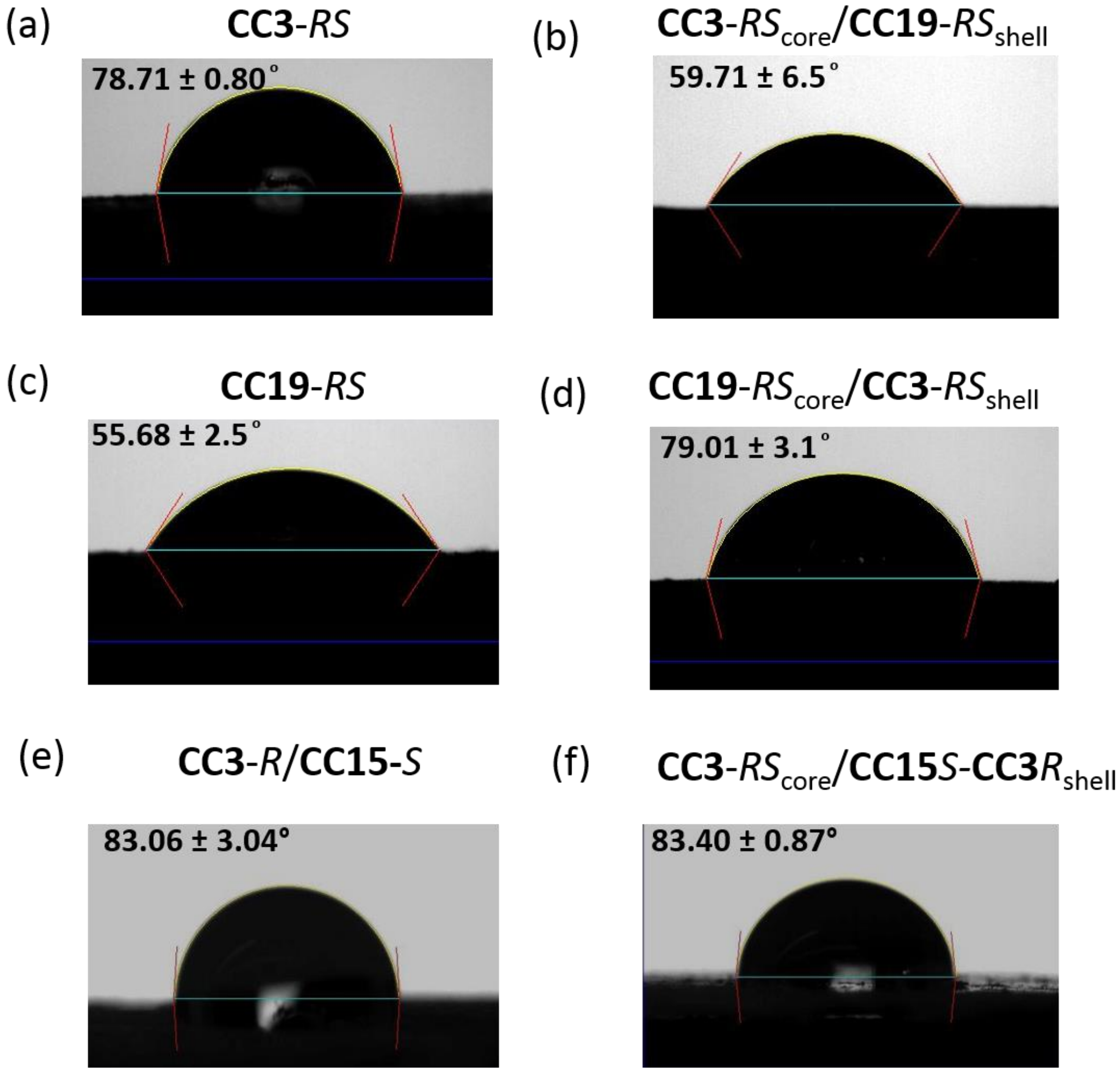

Figure S20. Contact angle measurement for (a) **CC3**-*RS*, (b) **CC3**-*RS*$_{\text{core}}$/**CC19**-*RS*$_{\text{shell}}$, (c) **CC19**-*RS*, (d) **CC19**-*RS*$_{\text{core}}$/**CC3**-*RS*$_{\text{shell}}$, (e) **CC3**-*R*/**CC15**-*S* and (f) **CC3**-*RS*$_{\text{core}}$/**CC15***S*-**CC3***R*$_{\text{shell}}$.

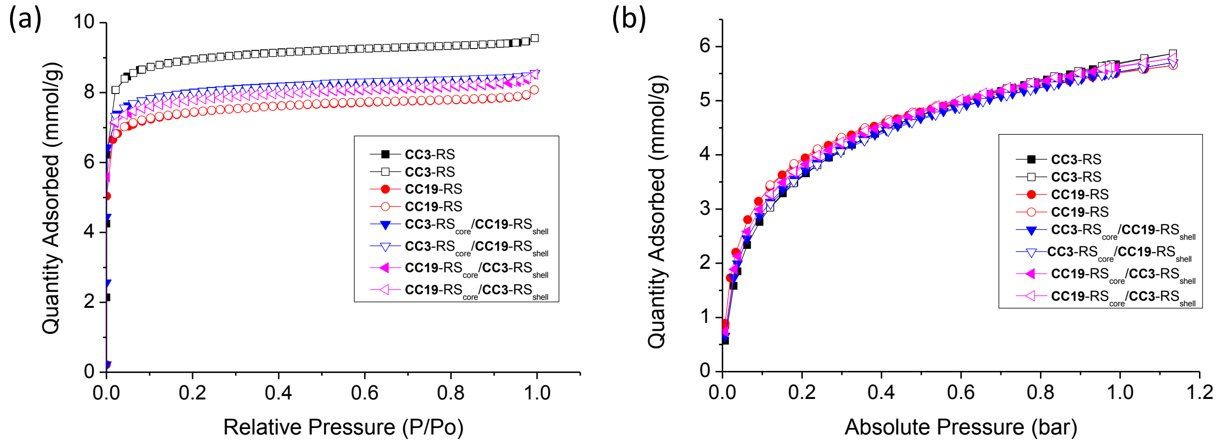

Figure S21. Gas sorption isotherms at 77 K for N_2 (a) and H_2 (b) for **CC3**-*RS*, **CC19**-*RS*, **CC3**-*RS*$_{core}$/**CC19**-*RS*$_{shell}$ and **CC19**-*RS*$_{core}$/**CC3**-*RS*$_{shell}$.

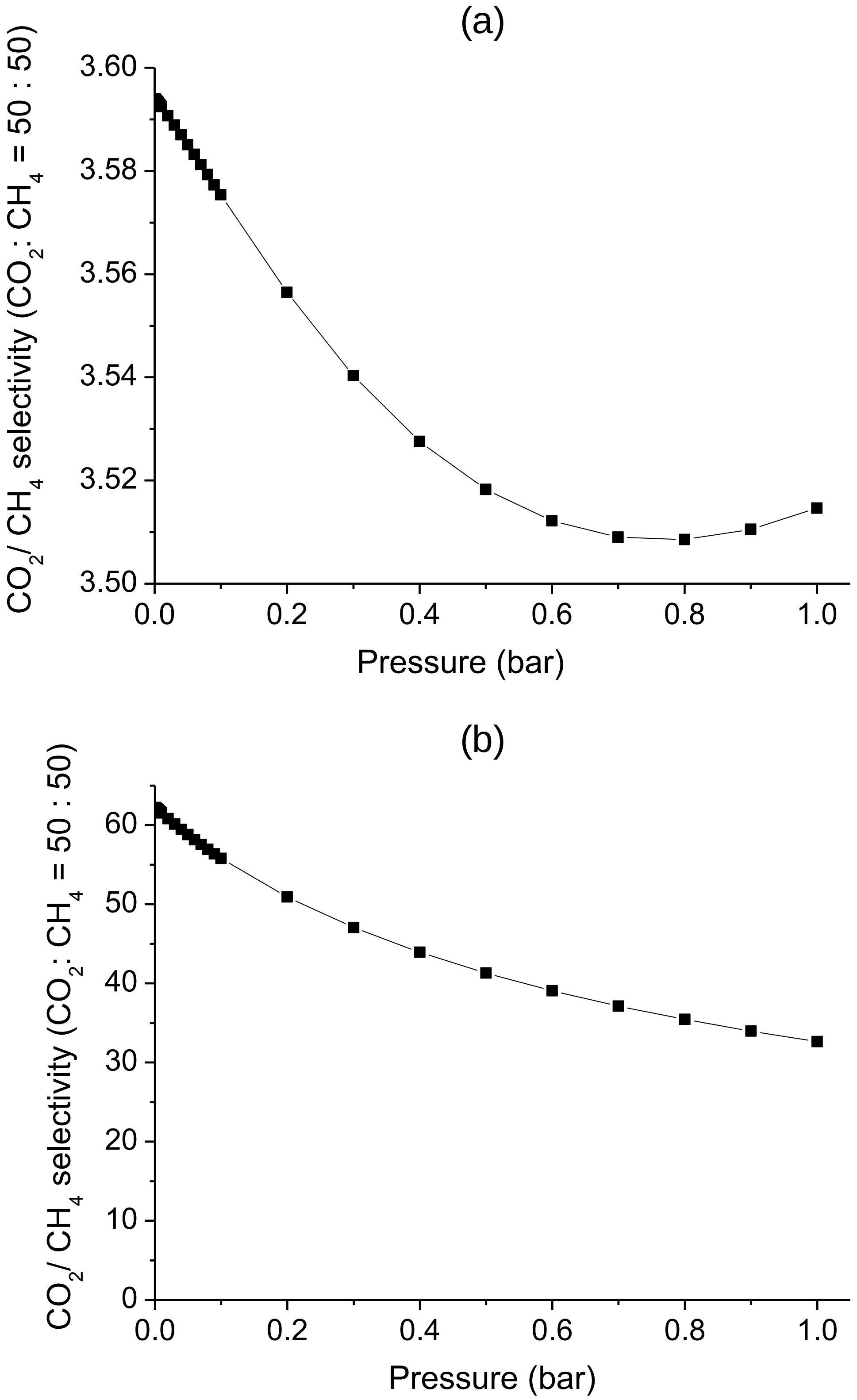

Figure S22. Pressure-dependent IAST selectivity of CO_2 over CH_4, as determined for equimolar mixtures using experimental single-component isotherms at 273 K: (a) **CC19**-*RS*$_{core}$/**CC3**-*RS*$_{shell}$; (b) **CC3**-*RS*$_{core}$/**CC19**-*RS*$_{shell}$.

Table S5. Gas sorption data of **CC3**-*RS*, **CC19**-*RS*, **CC3**-*RS*$_{core}$/**CC19**-*RS*$_{shell}$ and **CC19**-*RS*$_{core}$/**CC3**-*RS*$_{shell}$. Annotation of (a) indicates precipitation by mixing of pre-synthesised cages of opposite chirality, while annotation of (b) indicates formation by direct synthesis from racemic diamines. It can be noted that **CC19**-*RS* shows a higher CO_2 uptake than **CC3**-*RS* while at the same time a lower CH_4 uptake. However, this phenomenon seems to be amplified for the **CC3**-*RS*$_{core}$/**CC19**-*RS*$_{shell}$ crystals, for which the methane uptake is even further reduced. We have previously found that the presence of crystal defects can have a significant impact on the gas sorption properties of these materials.[3] It is likely that 'seeding' the growth of **CC19**-*RS* with **CC3**-*RS* crystals may lead to fewer defects, and therefore an even greater barrier to diffusion of methane. SA_{BET} was calculated from the N_2 isotherm in a relative pressure (P/P_0) range of 0.01-0.1.

	SA_{BET} / $m^2\ g^{-1}$	N_2 / mmol g^{-1} 77 K, P/P_0=0.998	H_2 / mmol g^{-1} 77 K, 1 bar	CO_2 / mmol g^{-1} 273 K, 1 bar	CH_4/ mmol g^{-1} 273 K, 1 bar
**CC3**-*RS*	598[a]	9.55[a]	5.87[a]	2.01[a]	1.85[a]
**CC19**-*RS*	500[a]	8.07[a]	5.65[a]	2.39[a]	1.34[a]
				1.39[b]	0.45[b]
**CC3**-*RS*$_{core}$/**CC19**-*RS*$_{shell}$ – 3 um	539	8.54	5.70	2.26	0.14
**CC19**-*RS*$_{core}$/**CC3**-*RS*$_{shell}$– 3 um	523	8.52	5.78	2.55	1.56

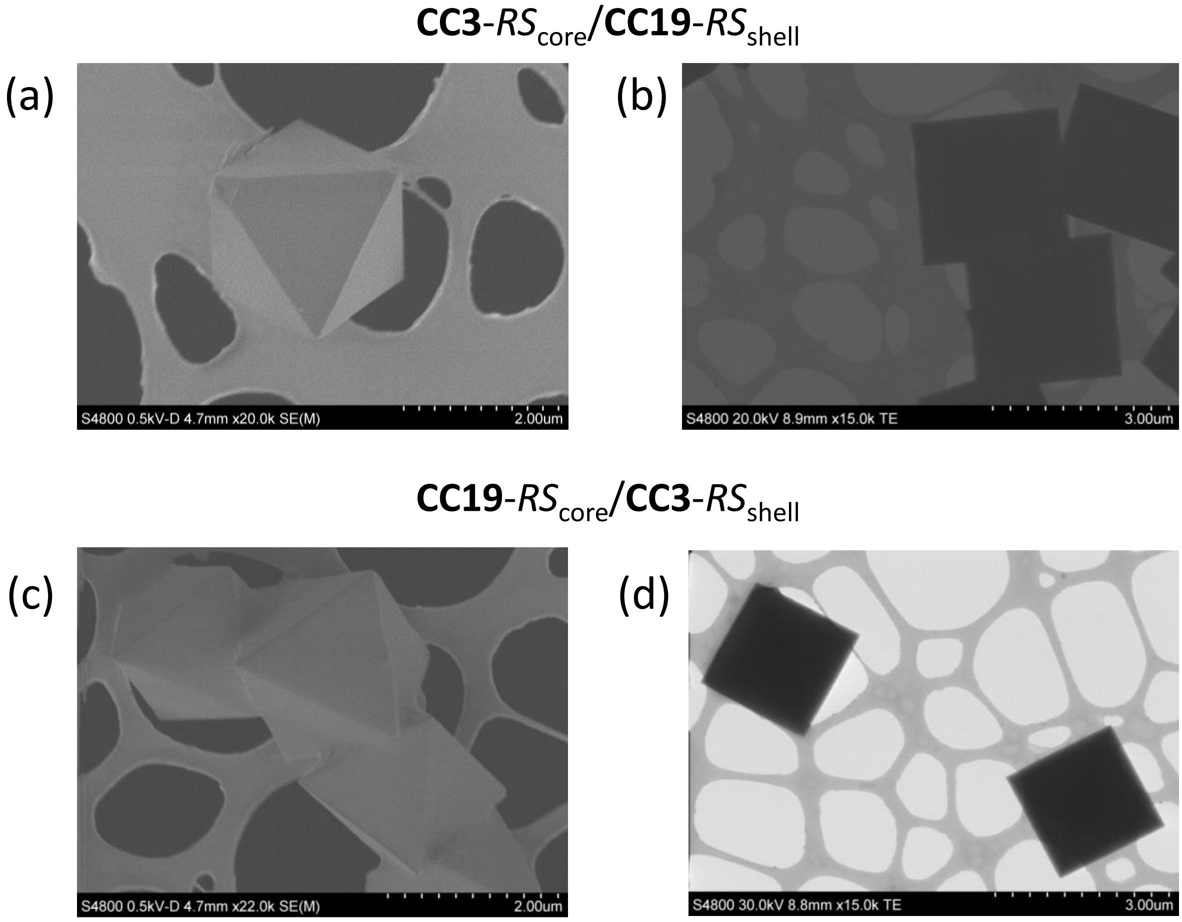

Figure S23. (a) STEM image for **CC3**-RS_{core}/**CC19**-RS_{shell} without gold coating, (b) TEM image for **CC3**-RS_{core}/**CC19**-RS_{shell}, (c) STEM image for **CC19**-RS_{core}/**CC3**-RS_{shell}, (d) TEM image for **CC19**-RS_{core}/**CC3**-RS_{shell}.

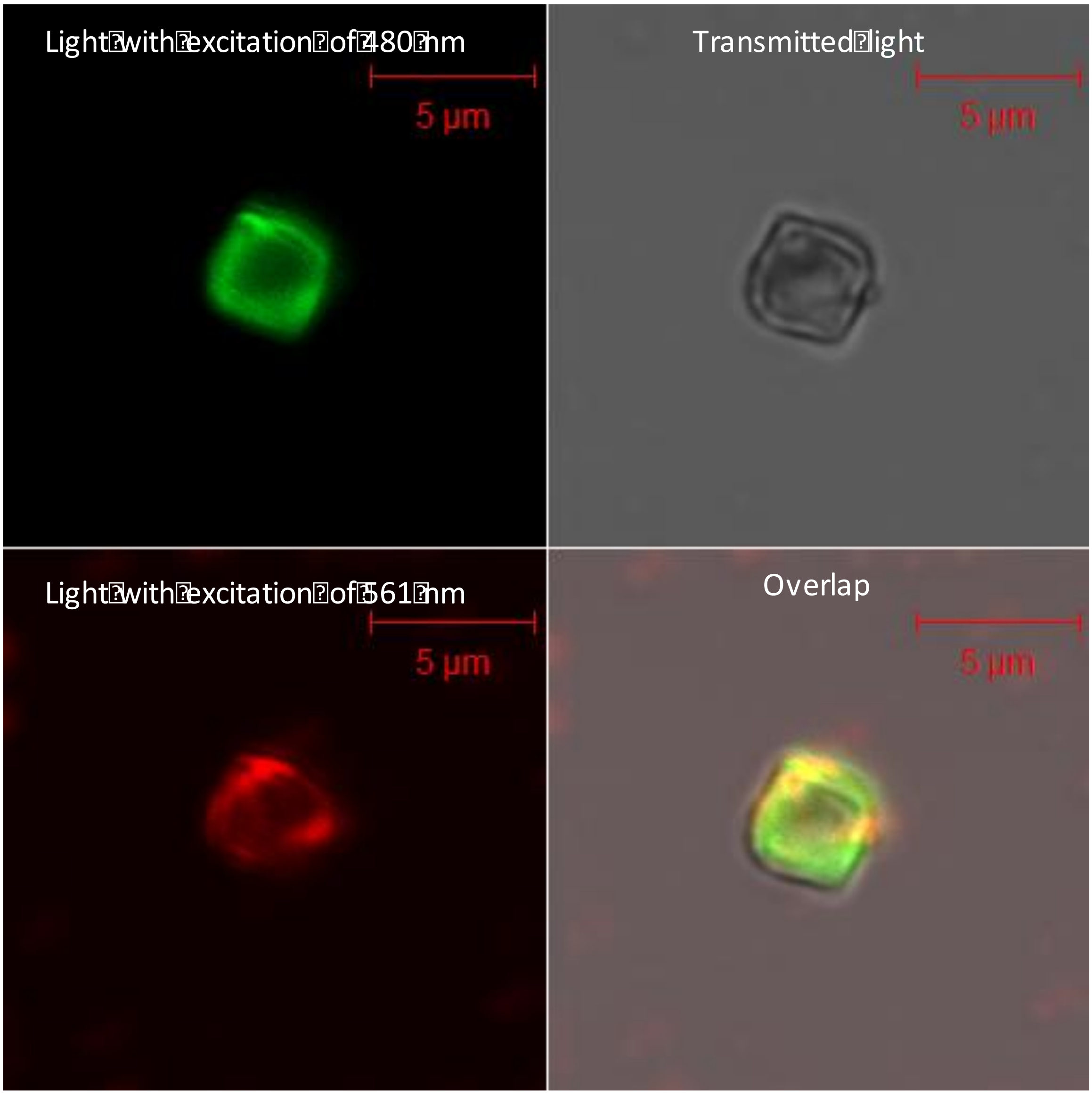

Figure S24. Confocal microscopy images for dye uptakes in the core-shell cage crystals. Dye molecules appear to be excluded from the inner core of the cage crystals.

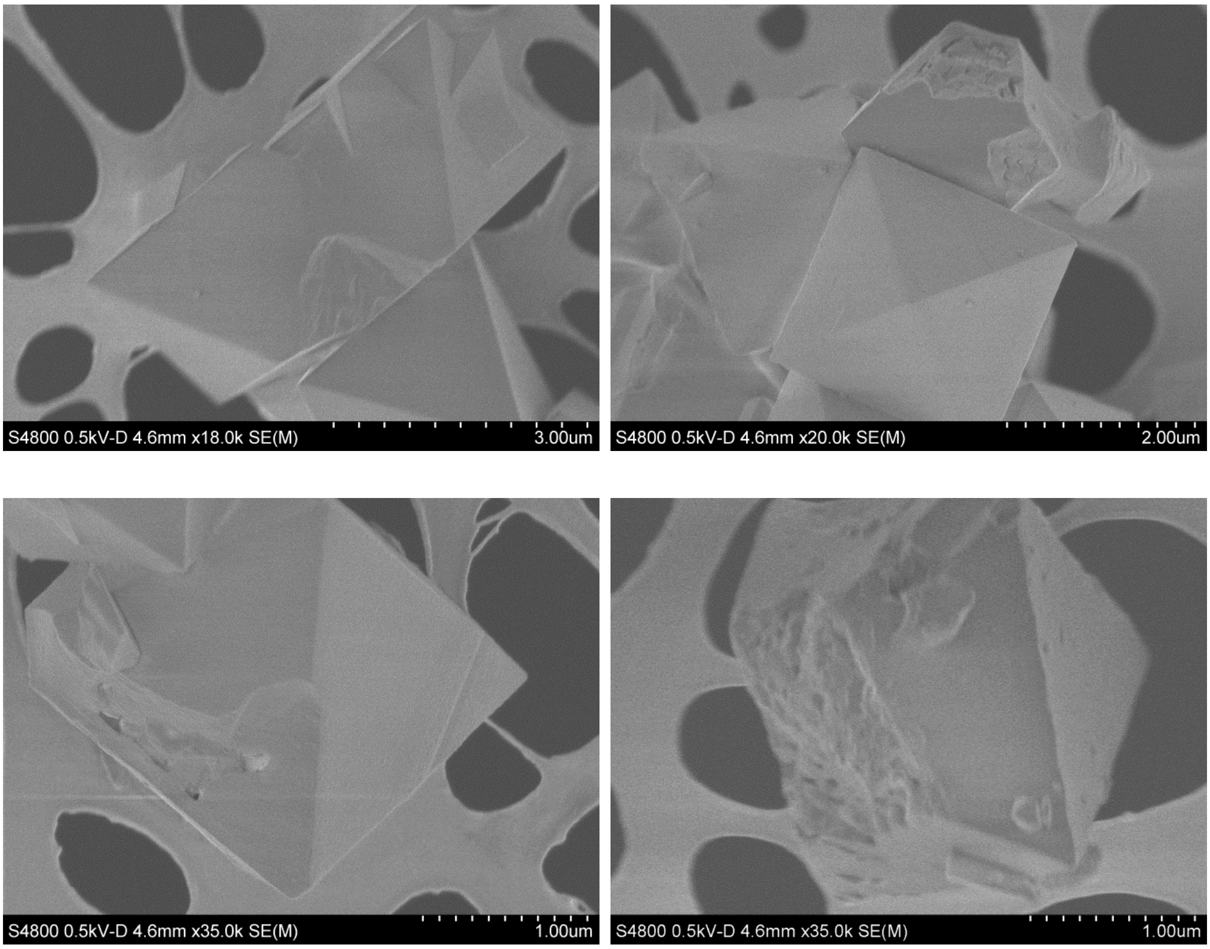

Figure S25. SEM images showing examples of damaged core-shell crystals in the sample.

Captions for movies

Movie S1: Z-stack of confocal images of **CC3**-*RS*$_{core}$/**CC19**-*RS*$_{shell}$. The confocal images were captured from the top surface to the bottom of crystal. The movie shows that the non-fluorescent inner core (**CC3**-*RS*) is covered by the fluorescent outer shell layer (**CC3**-*RS*).

Movie S2: A 3D structural model for **CC3**-*RS*$_{core}$/**CC19**-*RS*$_{shell}$ has been generated based on the z-stack of confocal analysis.

References

[1] (a) M. Petryk, J. Szymkowiak, B. Gierczyk, G. Spolnik, L. Popenda, A. Janiak, M. Kwit, *Org. Biomol. Chem.* **2016**, *14*, 7495-7499; (b) T. Tozawa, J. T. A. Jones, S. I. Swamy, S. Jiang, D. J. Adams, S. Shakespeare, R. Clowes, D. Bradshaw, T. Hasell, S. Y. Chong, C. Tang, S. Thompson, J. Parker, A. Trewin, J. Bacsa, A. M. Z. Slawin, A. Steiner, A. I. Cooper, *Nat. Mater.* **2009**, *8*, 973-978; (c) A. G. Slater, P. S. Reiss, A. Pulido, M. A. Little, D. L. Holden, L. Chen, S. Y. Chong, B. M. Alston, R. Clowes, M. Haranczyk, M. E. Briggs, T. Hasell, G. M. Day, A. I. Cooper, *ACS Cent. Sci.* **2017**, *3*, 734-742.

[2] T. Hasell, S. Y. Chong, K. E. Jelfs, D. J. Adams, A. I. Cooper, *J. Am. Chem. Soc.* **2012**, *134*, 588-598.

[3] A. A. Coelho, *TOPAS-Academic, v. 5; Coelho Software, Brisbane, Australia,* **2012**.

[4] T. J. Whittles, L. A. Burton, J. M. Skelton, A. Walsh, T. D. Veal, V. R. Dhanak, *Chem. Mater.* **2016**, *28*, 3718-3726.
